# Medical Outcomes, Quality of Life, and Family Perceptions for Outpatient vs Inpatient Neutropenia Management After Chemotherapy for Pediatric Acute Myeloid Leukemia

**DOI:** 10.1001/jamanetworkopen.2021.28385

**Published:** 2021-10-28

**Authors:** Kelly D. Getz, Julia E. Szymczak, Yimei Li, Rachel Madding, Yuan-Shung V. Huang, Catherine Aftandilian, Staci D. Arnold, Kira O. Bona, Emi Caywood, Anderson B. Collier, M. Monica Gramatges, Meret Henry, Craig Lotterman, Kelly Maloney, Amir Mian, Rajen Mody, Elaine Morgan, Elizabeth A. Raetz, Jeffrey Rubnitz, Anupam Verma, Naomi Winick, Jennifer J. Wilkes, Jennifer C. Yu, Brian T. Fisher, Richard Aplenc

**Affiliations:** 1 Division of Oncology, Children’s Hospital of Philadelphia, Philadelphia, Pennsylvania; 2 Department of Biostatistics, Epidemiology and Informatics, Perelman School of Medicine, University of Pennsylvania, Philadelphia; 3 Sidney Kimmel Medical College, Thomas Jefferson University, Philadelphia, Pennsylvania; 4 Department of Biomedical and Health Informatics, Children’s Hospital of Philadelphia, Philadelphia, Pennsylvania; 5 Department of Pediatrics, Division of Pediatric Hematology/Oncology, Stanford University, Palo Alto, California; 6 Children’s Healthcare of Atlanta, Emory University, Atlanta, Georgia; 7 Pediatric Hematology/Oncology, Children’s Hospital Boston, Boston, Massachusetts; 8 A.I. Dupont Hospital for Children, Nemours, Wilmington, Delaware; 9 University of Mississippi, Jackson; 10 Texas Children’s Hospital, Baylor College of Medicine, Houston; 11 Children’s Hospital of Michigan, Detroit; 12 Ochsner Medical Center for Children, New Orleans, Louisiana; 13 Children’s Hospital Colorado and the Department of Pediatrics, University of Colorado School of Medicine, Aurora; 14 Arkansas Children’s Hospital, Little Rock; 15 University of Michigan, Ann Arbor; 16 Ann & Robert H. Lurie Children’s Hospital of Chicago, Chicago, Illinois; 17 Stephen D. Hassenfeld Children’s Center for Cancer and Blood Disorders, New York, New York; 18 Department of Oncology, St Jude Children’s Research Hospital, Memphis, Tennessee; 19 Department of Pediatrics, Division of Pediatric Hematology/Oncology, University of Utah, Salt Lake City; 20 Department of Pediatric Hematology Oncology, University of Texas Southwestern Medical Center, Dallas; 21 Department of Pediatrics, University of Washington, Division of Hematology/Oncology, Seattle Children’s Hospital, Seattle; 22 Division of Pediatric Hematology Oncology, Rady Children’s Hospital San Diego, San Diego, California; 23 Division of Infectious Diseases, Children’s Hospital of Philadelphia, Philadelphia, Pennsylvania

## Abstract

**Importance:**

Pediatric acute myeloid leukemia (AML) requires multiple courses of intensive chemotherapy that result in neutropenia, with significant risk for infectious complications. Supportive care guidelines recommend hospitalization until neutrophil recovery. However, there are little data to support inpatient over outpatient management.

**Objective:**

To evaluate outpatient vs inpatient neutropenia management for pediatric AML.

**Design, Setting, and Participants:**

This cohort study used qualitative and quantitative methods to compare medical outcomes, patient health-related quality of life (HRQOL), and patient and family perceptions between outpatient and inpatient neutropenia management. The study included patients from 17 US pediatric hospitals with frontline chemotherapy start dates ranging from January 2011 to July 2019, although the specific date ranges differed for the individual analyses by design and relative timing. Data were analyzed from August 2019 to February 2020.

**Exposures:**

Discharge to outpatient vs inpatient neutropenia management.

**Main Outcomes and Measures:**

The primary outcomes of interest were course-specific bacteremia incidence, times to next course, and patient HRQOL. Course-specific mortality was a secondary medical outcome.

**Results:**

Primary quantitative analyses included 554 patients (272 [49.1%] girls and 282 [50.9%] boys; mean [SD] age, 8.2 [6.1] years). Bacteremia incidence was not significantly different during outpatient vs inpatient management (67 courses [23.8%] vs 265 courses [29.0%]; adjusted rate ratio, 0.73; 95% CI, 0.56 to 1.06; *P* = .08). Outpatient management was not associated with delays to the next course compared with inpatient management (mean [SD] 30.7 [12.2] days vs 32.8 [9.7] days; adjusted mean difference, −2.2; 95% CI, −4.1 to −0.2, *P* = .03). Mortality during intensification II was higher for patients who received outpatient management compared with those who received inpatient management (3 patients [5.4%] vs 1 patient [0.5%]; *P* = .03), but comparable with inpatient management at other courses (eg, 0 patients vs 5 patients [1.3%] during induction I; *P* = .59). Among 97 patients evaluated for HRQOL, outcomes did not differ between outpatient and inpatient management (mean [SD] Pediatric Quality of Life Inventory total score, 70.1 [18.9] vs 68.7 [19.4]; adjusted mean difference, −2.8; 95% CI, −11.2 to 5.6). A total of 86 respondents (20 [23.3%] in outpatient management, 66 [76.7%] in inpatient management) completed qualitative interviews. Independent of management strategy received, 74 respondents (86.0%) expressed satisfaction with their experience. Concerns for hospital-associated infections among caregivers (6 of 7 caregiver respondents [85.7%] who were dissatisfied with inpatient management) and family separation (2 of 2 patient respondents [100%] who were dissatisfied with inpatient management) drove dissatisfaction with inpatient management. Stress of caring for a neutropenic child at home (3 of 3 respondents [100%] who were dissatisfied with outpatient management) drove dissatisfaction with outpatient management.

**Conclusions and Relevance:**

This cohort study found that outpatient neutropenia management was not associated with higher bacteremia incidence, treatment delays, or worse HRQOL compared with inpatient neutropenia management among pediatric patients with AML. While outpatient management may be safe for many patients, course-specific mortality differences suggest that outpatient management in intensification II should be approached with caution. Patient and family experiences varied, suggesting that outpatient management may be preferred by some but may not be feasible for all families. Further studies to refine and standardize safe outpatient management practices are warranted.

## Key Points

**Question:**

What are the clinical outcomes and patient and family experiences associated with outpatient neutropenia management after intensive chemotherapy for pediatric acute myeloid leukemia (AML) compared with inpatient management?

**Findings:**

In this cohort study including as many as 554 pediatric patients, patients discharged to outpatient management during neutropenia did not experience higher bacteremia incidence, delays to subsequent courses, or worse health-related quality of life compared with those who received inpatient management. Most patients and families were satisfied with the discharge practice offered by the treating institution, but experiences varied, suggesting that outpatient management may not be appropriate for all families.

**Meaning:**

These findings suggest that outpatient management during neutropenia was a viable approach without excess risk for many patients and was preferred by some families.

## Teaser

This cohort study uses quantitative and qualitative methods to compare medical and quality of life outcomes among pediatric patients with acute myeloid leukemia receiving inpatient vs outpatient neutropenia management.

## Introduction

Pediatric patients with newly diagnosed acute myeloid leukemia (AML) receive multiple intensive chemotherapy courses, causing prolonged neutropenia and a high risk for life-threatening infections.^1,2^ In 2000, Children’s Cancer Group mandated hospitalization during neutropenia for patients in the CCG2961 clinical trial because of an initial 19% treatment-related mortality rate.^3^ This recommendation carried forward in all phase III Children’s Oncology Group (COG) AML clinical trials.^4,5^ Most centers in the US, Canada, and Europe follow this guideline, but some report outpatient management some or all of the time.^6,7,8^

Research comparing outpatient vs inpatient management has not included integrated assessments of medical outcomes, family preferences, and patient- and family-centered outcomes (PCOs). While adult AML studies suggest that outpatient management may be clinically safe,^9,10,11,12,13,14,15^ pediatric data are limited by conflicting results. A single-center study by Inoue et al^16^ reported similar rates of relapse and mortality for outpatient vs inpatient management of neutropenia. However, a retrospective multicenter observational study of administrative data found that outpatient management was associated with higher rates of antibiotic, vasopressor, and supplemental oxygen use.^6^ Additionally, data regarding patient and family preferences for inpatient vs outpatient management during neutropenia is limited to children with low-risk neutropenia,^17,18^ and preferences for management in children with AML have not been assessed, to our knowledge.

This study used a comprehensive mixed-methods design to compare medical outcomes, identify PCOs, assess health-related quality of life (HRQOL) and other identified PCOs, and describe the lived experience of outpatient and inpatient management after intensive AML chemotherapy. We hypothesized that outpatient management would be associated with an increased risk of bacteremia but higher quality of life.

## Methods

The study protocols were approved by institutional review boards at all participating sites. Consent requirements were waived for medical record extractions because the research met the criteria of 45 CFR 46.116(d). All patients who participated in interviews or completed questionnaires provided written informed consent. The analyses and reporting of the quantitative objectives followed the Strengthening the Reporting of Observational Studies in Epidemiology (STROBE) reporting guideline for observation cohort studies.

### Study Design

This cohort study used a multiphase mixed methods design (Figure). First, standardized medical record abstraction captured primary medical outcomes for pediatric patients with AML who received outpatient or inpatient neutropenia management. Next, semistructured interviews identified outcomes important to patients and their families.^19^ Patients receiving frontline chemotherapy were then prospectively invited to complete questionnaires measuring patient HRQOL and PCOs identified from the semistructured interviews. Lastly, the qualitative interviews provided depth of understanding and context for the quantitative findings.

**Figure.**
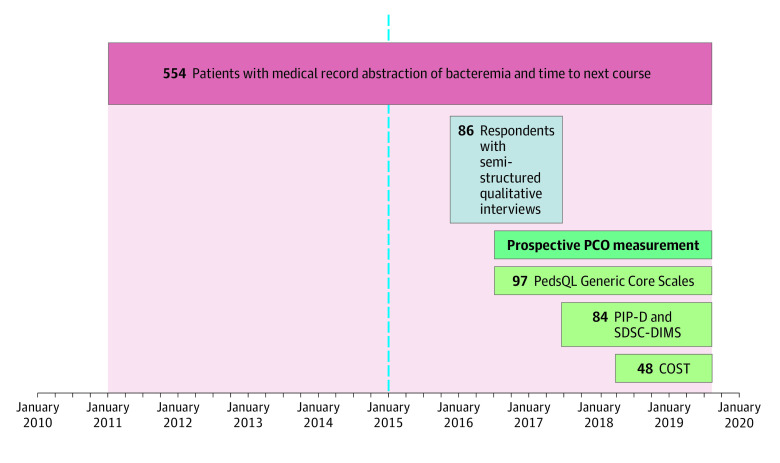
Study Schematic The vertical dotted line indicates the study start in January 2015; COST, Modified Comprehensive Score for Financial Toxicity; PCO, patient- and family-centered outcome; PedsQL, Pediatric Quality of Life Inventory; PIP-D, Pediatric Inventory for Parent–Difficulty; and SDSC-DIMS, Sleep Disturbance Scale-Disorders of Initiating and Maintaining Scale.

Patients were eligible for inclusion in the overall cohort if they had a diagnosis of AML and were aged younger than 19 years at initial diagnosis. Patients with acute promyelocytic leukemia and patients who received reduced intensity chemotherapy or only received a hematopoietic stem cell transplantation at the participating site were ineligible.

#### Medical Record Abstraction

Trained abstractors collected detailed data on demographics, diagnosis, treatment, antimicrobial prophylaxis, all inpatient and outpatient encounters, and all blood culture results from initial AML admission until recovery after the last frontline chemotherapy course for retrospectively and prospectively identified patients diagnosed from January 2011 to July 2019 at 17 pediatric institutions across the US. Institutions were surveyed for standard supportive care practices, including approach to systemic anti-infective prophylaxis, central line care, and antiseptic bathing protocols.

#### Qualitative Interviews

From November 2015 to February 2017, semistructured interviews were conducted at 9 pediatric institutions with patients and families experiencing either management strategy. Details about data collection and analysis have been previously reported in accordance with Consolidated Criteria for Reporting Qualitative Research (COREQ) reporting guideline.^19^ In addition to identifying PCOs for prospective assessment, interviews captured patient and family perceptions about the lived experience of neutropenia, including their satisfaction with their management strategy, impact on family life, and beliefs about risks and benefits of each approach.

#### Prospective Questionnaires

From June 2016 to May 2019, patients receiving frontline chemotherapy were prospectively invited to questionnaires administered at 2 time points during a single post–induction I chemotherapy course: within the period from first day to the last day of chemotherapy within the given course (baseline), and within the period from recovery of absolute neutrophil count to greater than 500 cells/uL (to convert to cells ×10^9^/L, multiply by 0.001) until start of next chemotherapy course (follow-up). Questionnaires collected socioeconomic information, acute Pediatric Quality of Life Inventory version 4.0 (PedsQL 4.0) Generic Core Scales,^20^ Pediatric Inventory for Parents-Difficulty (PIP-D) assessment,^21^ Sleep Disturbance Scale for Children-Disorders of Initiating and Maintaining Sleep (SDCS-DIMS) domain,^22^ and a modified Comprehensive Score for Financial Toxicity (COST) questionnaire.^23^

Based on expert recommendations on the use of established assessments, PedsQL and COST were administered at baseline and follow-up, whereas SDSC-DIMS and PIP-D were administered at follow-up only. SDSC-DIMS, PIP-D, and COST assessments were incorporated based on themes identified from analysis of the qualitative interviews^19^ and were implemented as the amended protocol was approved by the institutional review boards of participating sites. Therefore, not all assessments were administered to every participant who consented to participate.

### Exposure

Neutropenia management strategy, categorized as inpatient or outpatient, was the primary exposure. Patients discharged home within 3 days after course-specific chemotherapy completion were categorized as receiving outpatient management, even if they were subsequently readmitted. Patients meeting discharge eligibility criteria but remaining in inpatient care more than 3 days after chemotherapy completion were categorized as receiving inpatient management. The threshold defining outpatient management was chosen based on prior data from Getz et al^6^ on timing of initial discharge relative to course-specific chemotherapy completion and represents a period within which neutropenia recovery is improbable.

### Outcomes

Primary medical outcomes were course-specific bacteremia incidence and time to initiation of the next chemotherapy course. Bacteremia follow-up began 3 days after course-specific chemotherapy completion and continued until the earliest of death, absolute neutrophil count recovery to greater than 500 cells/uL, or initiation of subsequent chemotherapy course. First occurrence of bacteremia in each course was defined as a single positive blood culture result for a bacterial pathogen unless the bacterium was considered a common commensal organism by the National Healthcare Safety Network. For common commensals other than Viridans group streptococci, 2 positive cultures within 3 consecutive days were required for classification as bacteremia.

Course-specific mortality was a secondary medical outcome. Inpatient resource utilization and intensive care unit (ICU)–level requirements were additional secondary outcomes ascertained from the Pediatric Health Information Systems (PHIS) database for the subpopulation of patients treated at PHIS institutions.^24,25^

The primary PCO was HRQOL, measured using caregiver proxy responses to acute PedsQL 4.0 Generic Core Scales. Secondary PCOs included patient sleep disturbance, measured using the SDCS-DIMS^22^; parental stress, measured with the PIP-D^21^; and financial distress, evaluated with the modified COST.^23^

### Statistical Analysis

Analyses were restricted to post–induction I chemotherapy courses, an a priori decision based on prior data that discharge during the first course is rare even at institutions where outpatient management is standard practice. Analyses at each treatment course were restricted to patients considered discharge-eligible to address potential confounding. Patients were considered discharge-eligible if there was no evidence of microbiologically documented infection, fever, or ICU-level requirements within 3 days of the last dose of systemic chemotherapy in the given course. Log-binomial regression estimated risk ratios (RRs) comparing incidence of bacteremia, and ICU-level care (restricted to PHIS sites) by outpatient vs inpatient management. Linear regression models compared times to next course. Analyses were conducted for each course separately, and general estimating equations accounted for nonindependence of observations from patients within an institution. Analysis of covariance compared PedsQL scores while controlling for baseline values. Linear regressions compared follow-up PIP-D scores and change in COST scores, and generalized linear models with γ distribution compared SDSC-DIMS scores.

Control for confounding was accomplished through adjustment for propensity score quintiles as well as any remaining unbalanced patient- or hospital-level confounders. Propensity scores were derived from predicted probabilities estimated from regressions of outpatient vs inpatient management conditional on baseline factors determined from bivariate analyses to be associated (*P* < .20) with both exposure and outcome of interest, or associated only with outcome.

SAS statistical software version 9.3 (SAS Institute) was used for all statistical analyses. *P* values were 2-sided, and statistical significance was set at *P* < .05. Additional details are presented in the eMethods in the Supplement. Final analyses of medical outcomes were performed from August 2019 to January 2020, and PHIS resource use was analyzed from November 2019 to January 2020. PCOs were analyzed from August 2019 to February 2020.

## Results

### Medical Outcomes

The full abstraction cohort included 610 patients treated during the study periods at each contributing site. Of these patients, 554 (90.8%) met early discharge–eligibility criteria in at least 1 course and contributed a total of 1196 post–induction I courses (eFigure 1 in the Supplement). Table 1 presents distributions of patient and hospital characteristics for the induction II study population of 493 patients, including 114 patients (23.1%) who received outpatient management and 379 patients (76.9%) who received inpatient management. Recognizing some course-specific variability, patients discharged to outpatient management were less likely to have high-risk AML, receive *Pneumocystis jiroveci* pneumonia (PJP) prophylaxis, or to receive treatment at institutions practicing line-lock therapy compared with those who received inpatient neutropenia management. They were also more likely to be aged 2 years or older at diagnosis, treated on a St. Jude trial, publicly insured or have undocumented insurance status, and treated at an institution that used antiseptic bathing (eTable 1 in the Supplement).

**Table 1.** Baseline Demographic, Clinical, and Hospital-Level Characteristics for Outpatient vs Inpatient Management, Induction II

Characteristic	Patients at induction II, No. (%)		*P* value
	Outpatient (n = 114)	Inpatient (n = 379)	
Sex			
Female	53 (46.5)	192 (50.7)	.44
Male	61 (53.5)	187 (49.3)	
Age at diagnosis, y			
0 to 1	18 (15.8)	145 (38.3)	<.001
2 to 10	45 (39.5)	107 (28.2)	
≥11	51 (44.7)	127 (33.5)	
Race			
Asian	11 (9.7)	26 (6.9)	<.001
Black	17 (14.9)	74 (19.5)	
White	52 (45.6)	224 (59.1)	
Other^a^	33 (29.0)	44 (11.6)	
Not recorded in EMR	1 (0.9)	11 (2.9)	
Hispanic ethnicity	29 (25.4)	77 (20.3)	.24
Insurance at course start			
Any private	46 (40.4)	181 (47.8)	.04
Public only or uninsured	59 (51.8)	174 (45.9)	
Other	4 (3.5)	21 (5.5)	
Not recorded in EMR	5 (4.4)	3 (0.80)	
Year of diagnosis			
2011-2013	34 (29.8)	100 (26.4)	.19
2014-2016	54 (47.4)	159 (42.0)	
2017-2019	26 (22.8)	120 (31.7)	
AML diagnosis type			
De novo	110 (96.5)	358 (94.5)	.47
Secondary or from TMD	4 (3.5)	21 (5.5)	
Risk classification			
Low	83 (72.8)	253 (66.8)	.03
Intermediate	10 (8.8)	17 (4.5)	
High	21 (18.4)	109 (28.8)	
Trial enrollment			
No	64 (56.1)	238 (62.8)	.002
Yes (COG trial)	32 (28.1)	120 (31.7)	
Yes (St Jude trial)	18 (15.8)	21 (5.5)	
Chemotherapy regimen			
ADE	95 (83.3)	246 (64.9)	.002
AE	0 (0)	6 (1.6)	
MA	13 (11.4)	98 (25.9)	
HD AraC	2 (1.8)	5 (1.3)	
Other	4 (3.5)	4 (3.5)	
Central line type at start of course			
Tunneled catheter	76 (66.7)	257 (67.8)	.42
Implanted port	8 (7.0)	30 (7.9)	
PICC	29 (25.4)	92 (24.3)	
No central line	1(0.9)	0 (0)	
Any PJP coverage	100 (87.7)	369 (97.4)	<.001
Any antibacterial prophylaxis	43 (37.7)	166 (43.8)	.28
Broad gram-positive coverage	41 (36.0)	163 (43.0)	.19
Broad gram-negative coverage	41 (36.0)	161 (42.5)	.23
Antipseudomonal coverage	40 (35.1)	159 (42.0)	.23
Broad anaerobic coverage	8 (7.0)	13 (3.4)	.11
MRSA coverage	31 (27.2)	120 (31.7)	.36
Hospital anti-infective practices			
Any antibactierial prophylaxis	47 (41.2)	170 (44.9)	.49
Line lock therapy	23 (20.2)	113 (29.8)	.04
Antibiotic bathing	79 (69.3)	237 (62.5)	.19

Abbreviations: ADE, cytarabine, daunorubicin, and etoposide; AE, cytarabine and etoposide; AML, acute myeloid leukemia; COG, Children’s Oncology Group; EMR, electronic medical record; HD AraC, high-dose cytarabine with or without asparaginase; MA, mitoxantrone and cytarabine; MRSA, methicillin-resistant *Staphylococcus aureus*; PICC, peripherally inserted central catheter; PJP, *Pneumocystis jiroveci* pneumonia; TMD, transient myeloproliferative disorder.

^a^ Other ace category includes American Indian or Alaska Native, Native Hawaiian or Pacific Islander, and those recorded as other race.

The proportion of patients who received outpatient management varied by chemotherapy course ranging from 7 of 44 patients (15.9%) in intensification III to 104 of 374 patients (27.8%) in intensification I (eFigure 2 and eTable 2 in the Supplement). Readmission rates after discharge to outpatient management were high across courses, with median time to first readmission ranging from 7 to 9 days (eTable 2 in the Supplement).

Incidence of bacteremia during postchemotherapy neutropenia increased with each course, from 103 patients (20.9%) during induction II to 123 patients (43.2%) during intensification II. Course-specific differences in incidence of bacteremia for outpatient vs inpatient management were not statistically significant (Table 2). Times to the next course ranged from 31 to 40 days overall. Differences in time-to-next course varied by course, but was only statistically significant for outpatient management compared with inpatient management in induction II (difference, −3.1 [95% CI, −5.2 to −1.0] days; *P* = .003) (Table 2). Course-specific mortality rates were low overall, but significantly higher in intensification II for patients who received outpatient management compared with those who received inpatient management (3 patients [5.4%] vs 1 patient [0.5%]; *P* = .03) (eTable 3 in the Supplement).

**Table 2.** Comparisons of the Incidence of Bacteremia During Postchemotherapy Neutropenia and Time to Next Frontline Chemotherapy Course for Outpatient vs Inpatient Neutropenia Management

Course	Overall	Outpatient	Inpatient	Crude value	*P* value	Adjusted value	*P* value
**Bacteremia, No. (%)**							
Induction II	103 (20.9)	22 (19.3)	81 (21.3)	0.90 (0.59 to 1.38)^a^	.64	0.85 (0.53 to 1.36)^a^^,^^b^	.50
Intensification I	105 (28.1)	27 (26.0)	78 (28.9)	0.90 (0.62 to 1.31)^a^	.58	0.76 (0.51 to 1.12)^a^^,^^b^	.16
Intensification II	123 (43.2)	18 (32.1)	105 (45.8)	0.70 (0.47 to 1.05)^a^	.09	0.74 (0.47 to 1.16)^a^^,^^b^	.19
Intensification III	1 (2.3)	0 (0)	1 (2.7)	NE		NE	
Across courses	332 (27.8)	67 (23.8)	265 (29.0)	0.79 (0.62 to 1.03)	.07	0.73 (0.56 to 1.06)	.08
**Time to next chemotherapy course, mean (SD)**							
Induction II	30.6 (9.1)	27.8 (7.6)	31.5 (9.4)	−3.7 (−5.7 to −1.7)^c^	.002	−3.1 (−5.2 to −1.0)^c^^,^^d^	.003
Intensification I	33.7 (10.7)	33.6 (14.4)	33.7 (8.7)	−0.1 (−2.9 to 2.7)^c^	.94	−1.0 (−4.2 to 2.1)^c^^,^^d^	.51
Intensification II	40.1 (15.2)	44.0 (26.5)	39.4 (13.0)	4.6 (−8.3 to 17.4)^c^	.49	−1.5 (−12.8 to 9.8)^c^^,^^d^	.79
Across courses	32.3 (10.4)	30.7 (12.2)	32.8 (9.7)	−2.4 (−4.4 to −0.4)^c^	.02	−2.2 (−4.1 to −0.2)^c^^,^^d^	.03

Abbreviation: NE, not estimable.

^a^ Presented as risk ratio (95% CI).

^b^ Adjusted for propensity score quintile. The propensity score model included age, race, insurance, risk classification, clinical trial enrollment, PJP coverage, Broad Gram positive coverage, Broad Gram negative coverage, and hospital-level anti-infective practices.

^c^ Presented as difference (95% CI).

^d^ Adjusted for propensity score quintile. The propensity score model for included age, chemotherapy regimen, central line type, *Pneumocystis jiroveci* pneumonia coverage, broad gram-positive coverage, broad gram-negative coverage, and hospital-level anti-infective practices.

### Subpopulation Comparison of ICU Care Requirements

Among 456 patients treated at PHIS-contributing institutions, those discharged to outpatient management were more likely to require ICU-level care compared with those who received inpatient management during intensification I (10 patients [13.0%] vs 20 patients [7.8%]; adjusted RR: 2.50 [95% CI, 1.03 to 6.06]) (eTable 4 in the Supplement). Relative differences in ICU-level requirements for outpatient vs inpatient management were not statistically significant during induction II (4 patients [4.6%] vs 9 patients [2.6%]; adjusted RR, 2.16 [95% CI, 0.48 to 9.62]) or intensification II (6 patients [10.9%] vs 20 patients [10.2%]; adjusted RR, 0.80 [95% CI, 0.32 to 1.99]).

### HRQOL and PCOs 

eFigure 3 in the Supplement depicts the study population who completed HRQOL and secondary PCO assessments. eTables 5-7 in the Supplement present distributions of patient and caregiver characteristics by management strategy. Comparisons of HRQOL and other secondary PCOs are presented in Table 3. Mean PedsQL Generic Core scores were low and did not differ for outpatient management (22 patients; mean [SD] score, 70.1 [18.9]) and inpatient management (75 patients; mean [SD] score: 68.7 [19.4]). Results were similar for physical and psychosocial health subscales.

**Table 3.** Crude and Adjusted Comparisons of Patient Health-Related Quality of Life, Patient Sleep Disturbance, Parental Stress, and Parental Financial Distress for Outpatient vs Inpatient Management

Outcome	Mean (SD)		Crude mean difference (95% CI)	*P* value	Adjusted mean difference (95% CI)	*P* value
	Outpatient	Inpatient				
PedsQL 4.0^a^						
Generic Core Scales total score	70.1 (18.9)	68.7 (19.4)	1.4 (−7.8 to 10.7)	.76	−2.8 (−11.2 to 5.6)	.56
Psychosocial health subscore	73.1 (18.7)	70.8 (18.2)	2.3 (−6.4 to 11.1)	.60	−2.4 (−10.3 to 5.4)	.54
Physical health subscore	64.7 (23.6)	63.8 (23.4)	0.9 (−11.5 to 13.2)	.89	−2.2 (−13.7 to 9.4)	.71
Patient sleep disruption^b^	56.1 (10.1)	61.7 (12.0)	−5.7 (−11.1 to −0.40)	.04	−6.0 (−11.9 to −0.1)	.05
Parental stress^c^	102.2 (30.5)	115.9 (28.8)	−13.8 (−27.9 to 0.40)	.06	−14.4 (−29.0 to 0.22)	.05
Parental financial distress, mean (SD), change^d^	0.25 (4.2)	−0.03 (5.9)	0.28 (−3.9 to 4.5)	.90	0.05 (−4.2 to 4.3)	.98

Abbreviation: PedsQL, Pediatric Quality of Life Inventory.

^a^ Higher scores reflect better patient health-related quality of life. Adjusted for baseline score and propensity score quintile. Propensity score model includes patient age, race, ethnicity, insurance, chemotherapy regimen, and caregiver education.

^b^ Assessed with Sleep Disturbance Scale-Disorders of Initiating and Maintaining Scale score. Higher scores indicate more sleep disturbance. Adjusted for propensity score quintile and hospital-level antimicrobial prophylaxis.

^c^ Assessed with Pediatric Inventory for Parent–Difficulty assessment. Higher scores indicate more parenting stress related to difficulty caring for a child with cancer. Adjusted for propensity score quintile and hospital-level antimicrobial prophylaxis.

^d^ Assessed with Modified Comprehensive Score for Financial Toxicity (COST) score change. Data presented are the change in COST scores from baseline to follow-up assessment. Measure of association reflect the mean difference in change scores between outpatient and inpatient such that positive differences suggest worse financial distress among the inpatient management group. Adjusted for propensity score quintile.

Patient sleep disturbance and parental stress (including 22 patients in outpatient management and 62 patients in inpatient management), and financial distress (including 8 patients in outpatient management and 40 patients in inpatient management) were measured for smaller subsets of the overall prospective study population (eFigure 3, eTable 6, and eTable 7 in the Supplement). Patients in outpatient management had significantly lower SDSC-DIMS T scores, indicating better sleep initiation and maintenance, than those in inpatient management (Table 3). In addition, while a larger proportion of patients in inpatient management experienced scores consistent with pathological disorders of sleep initiation and maintenance (16 patients [22.2%] vs 2 patients [9.1%]; *P* = .22), this difference was not statistically significant.

Although point estimates for parental stress as measured by total PIP-D scores were lower for caregivers of children in outpatient management compared with caregivers of children in inpatient management, the difference was not statistically significant (Table 3). More than 95% of caregivers reported financial difficulties regardless of discharge strategy (eTable 8 in the Supplement). No significant difference in the change in COST scores was reported (Table 3). While many financial expenses were similar by management strategy, difficulties paying for mortgage or rent were more commonly reported for outpatient management than inpatient management (13 of 22 parents [59.1%] vs 16 of 62 parents [25.4%]; *P* = .008) (eTable 8 in the Supplement).

### Patient and Caregiver Perceptions

We interviewed 86 respondents (32 children and 54 caregivers) from 57 families with a child who received AML chemotherapy. Of these 57 families, 39 received inpatient management and 18 received outpatient management. Exemplar quotes demonstrating key qualitative findings are presented in Table 4.

**Table 4.** Exemplar Quotes Demonstrating Key Qualitative Findings

Theme	Quote	Respondent	Neutropenia management strategy
Hospital perceived to be safer for the child	At the hospital, he got the help he needed, if things go downhill. There was a point where he was done with his chemo[therapy] and his ANC went down as expected and then started climbing up. He was feeling just fine. He was eating. He was drinking. He was playing. And then [snaps fingers] a fever.…He went to bed 1 night totally fine and woke up with a 104[° F] fever just a few hours later. It’s just that quick, within 4 hours quick. And he was on the verge of going to the ICU. So yeah, now think of your child being at home, sitting on the couch and that happens? Do you call an ambulance? Do you get in the car and run stop signs to get to the hospital? Or would you rather already be at the hospital?	Father	Inpatient
Home perceived to be cleaner and safer for the child	Pretty much, I can keep his bedroom cleaner than the hospital room. To be honest with you, some days, I come here [to the hospital] and his floor is gross, and I’m like, “What the heck is going on? Can you please send environmental up here to clean his floor? It’s disgusting.” It’s just disgusting. Then, he caught, each time he was here [at the hospital], he caught *C. difficile*, which I know he probably wouldn’t have caught if he was at home, because it’s a hospital-borne illness, and that was a really tough time for him too, going through that and being treated for it, bad stomach cramping. He’s like balled up in knots. He can’t eat because he’s running to jump up, and he has to have a commode by his bed.	Mother	Inpatient
Patient preference for inpatient management owing to home instability	I was at Children’s Hospital for 9 months. The first month or two, maybe the first month and a half, it wasn’t—it didn’t feel like home, but once I got used to it, you know, being around the environment and the sweet people and the nurses and doctors and everything and knew around the place, it was okay. It felt like I was home...I stopped noticing that I was in the hospital and I just felt like I was at home and, you know, like I was around sweet people, like I don’t have to worry about yelling anymore, I don’t have to worry about people lying, I don’t have to worry about nothing bad at all. It was all good vibes and good things.	Patient	Inpatient
	To be honest, after going through it all, I actually would’ve rather stayed at the hospital, because the noise at home, after being perfectly quiet in the hospital room and the only thing happening was the beeping of the pump. That doesn’t match how many kids are in our house yelling and hitting each other daily. So I would actually kind of rather stayed—because the noise itself just caused such a lot of stress for me, because I just hated that, compared to all the nice, perfectly quiet hospital room.	Patient	Inpatient
Home perceived as closer to family and normal life	The best part was just the comfort of home, just having her own bed and privacy and stuff like that. Being around family members, it was like a refresher. Even though you were scared to death, it was a refresher to go home, be normal for a while, be in our own home with our own stuff.	Mother	Outpatient
Home perceived as more stressful because of lack of support	I would have much rather been in the hospital the whole time. I loved it. I loved it because it was stressless. It’s better than being home [be]cause when I was home I would always shiver. I was so scared. But when I was there, I never shivered. When I was home, I didn’t get any help and I couldn’t sleep [be]cause I was always watching her. That’s why I liked going to the hospital more. I could sleep in the hospital. And that was on a couch. I’d find that room to be like a castle.	Mother	Outpatient

Abbreviations: ANC, absolute neutrophil count; ICU, intensive care unit.

#### Inpatient Management

A total of 57 respondents who experienced inpatient management (86.4%; 32 caregivers, 25 children) expressed satisfaction with hospitalization for the duration of neutropenia. Respondents perceived the hospital to be the safest place for their child owing to its close proximity to emergency care, the reassurance of constant medical surveillance, and a perceived lower risk of infection. They noted that prolonged hospitalization could be difficult, but positive aspects included engagement with Child Life Service staff, meaningful relationships with clinical caregivers, and the perception that the hospital was a safe space where children did not have to explain their illness to others.

A total of 9 respondents (13.6%; 7 caregivers and 2 children) who experienced inpatient management were dissatisfied. Both dissatisfied children (100%) cited family separation and 6 dissatisfied caregivers (85.7%) cited a belief that the hospital was riskier with respect to infection. Four children of the 7 caregivers dissatisfied with inpatient management preferred the hospital. These children perceived their home environment differently than their caregivers, describing it as stressful and chaotic.

#### Outpatient Management

A total of 20 respondents (85.0%; 12 caregivers and 5 children) who experienced home management were satisfied with their experience. They emphasized the psychosocial benefits for the child. Three respondents (15.0%; all caregivers) dissatisfied with outpatient management found caring for a neutropenic child at home to be very stressful owing to lack of support from family, challenges of managing infection risk, and anxiety about monitoring their child’s health.

## Discussion

This mixed-methods cohort study reports multiple medical and PCOs associated with management of patients receiving AML chemotherapy. Notably, pediatric patients with AML who were discharged to outpatient management did not have higher rates of bacteremia or delays in progression to subsequent treatment courses compared with those who received inpatient management. While the mortality rates overall were generally low, mortality in intensification II was higher in patients receiving outpatient management. Patient HRQOL was remarkably low for both groups, with patient sleep disruption better and parental stress scores lower (although not statistically significant) for outpatient management. Although the overall degree of financial distress during treatment was similar between management strategies, stress regarding making mortgage or rent payments was more prevalent in families experiencing outpatient management. Qualitative interviews revealed that most patients and families preferred the discharge strategy recommended by their treating institution.

These results align with prior studies demonstrating low mortality rates,^16^ fewer days of febrile neutropenia, less antimicrobial use,^26^ and higher ICU resource utilization for outpatient management.^6^ Work in other pediatric cancer populations has also demonstrated sleep disturbances in children receiving inpatient care,^27^ and low HRQOL has been similarly described in pediatric AML.^28^

These findings have multiple implications for the care of children with AML. First, these data support outpatient management for select patients in post–induction I chemotherapy courses except intensification II. Outpatient management, as presaged in the qualitative data, may be associated with a lower rate of bacteremia and less sleep disruption, and potentially lower stress. For many families, these benefits could lead to a strong preference for outpatient management. However, the increased need for ICU-level support in patients who received outpatient management, which may reflect more severe infectious complications, may sway some families to prefer inpatient management. This preference may be heightened in families who view outpatient management as inherently more stressful and less safe than inpatient management.

The rarity of mortality events in intensification II makes definitive conclusions regarding the safety of outpatient management in that course challenging. However, given extensive external data documenting increased toxic effects and mortality in intensification II,^5,29^ outpatient management in that course should be approached with particular caution.

Importantly, no single end point reported in this study can determine the optimal discharge strategy for an individual patient and their family. Rather, information from each data domain must be assessed by the clinical care team and integrated into nuanced discussions with patients and families. Some of these assessments typically are performed by nonclinicians, such as social workers, and require careful discussions of complex topics, such as physical or financial security. Close collaboration between medical and nonmedical staff is essential to determining the most effective discharge strategy for an individual patient. Discrepancies in preferences between patient and caregiver will need particularly close attention and careful management in determining the optimal approach. The coordinated effort needed to personalize neutropenia management for an individual patient may exceed the efforts needed to personalize therapy based on AML molecular characteristics.

These findings also have broader implications for children receiving chemotherapy and their families. These results clearly demonstrate the diverse experiences and preferences of patients and families during cancer care. Such heterogeneity is assuredly present across pediatric cancers and the processes needed to personalize AML hospital management will likely be applicable to multiple other supportive care practices. The remarkable breadth of information obtained from the mixed-methods approach would likely be reproduced in other pediatric cancer care questions. Moreover, the inclusion of patient and family voices directly in the research process provided an invaluable perspective that arguably should be included in all clinical evaluations that seek to provide a definitive care recommendation.

### Limitations

Despite the multisite, mixed-methods approach, this study has limitations. The multi-center design was not able to overcome sample size limitations owing to the rarity of pediatric AML. While appropriately powered for the analyses of the primary medical outcomes, analyses of the secondary prospectively measured outcomes are based on small numbers. Additionally, course-specific comparisons of mortality in intensification II were based on very rare events. We sought to address these limitations with cautious interpretation of individual comparisons and careful evaluation of magnitudes of association and CIs.^30,31^ Still, further evaluations of these prospectively measured PCOs within larger cohorts are warranted. Second, the manual health record abstraction, while extensive, did not include fungal infections or information required to enable systematic grading of bacteremia and sepsis. However, our secondary analyses of resource utilization aimed to provide additional context to the primary bacteremia comparison specifically related to relative overall acuity of patients who were discharged to outpatient management and those who were not. Future studies are warranted to identify potential mechanisms for the observed difference in ICU-level care requirements. Third, all patients were enrolled at US centers. The marked disparities in resources available to patients and families may limit direct generalizability of our findings to countries with national health insurance and strong social safety nets. Fourth, while we accounted for hospital-level infection control and central line management practices and use statistical methods to account for hospital-level clustering, there is a possibility for residual confounding by unmeasured or poorly measured factors, such as specific psychosocial or logistical factors considered by the institutions where standard practice was early discharge to outpatient management. Furthermore, 2 qualitative method limitations should be recognized. Despite focused efforts to include a diverse range of participants, interviewed patients and families may differ systematically from those declining interviews. Since demographic data on nonparticipants were not collected, an assessment of potential bias implications is not possible. However, this concern is partially mitigated by the large number and wide geographic distribution of interviewed patients and families. Additionally, perspectives of treating clinicians were not captured either in interviews or surveys; this is an important limitation that will need to be addressed in subsequent implementation studies.

## Conclusions

This cohort study found that outpatient neutropenia management for pediatric AML may be undertaken safely for select patients and may be less burdensome to patients and families than inpatient management. However, not all chemotherapy courses may be amenable to outpatient management, given current supportive care and health care access in the US, and not all families will experience outpatient management as a preferred strategy. The combined use of medical outcomes, PCOs and qualitative interview data provides dramatically richer and more nuanced data to guide clinicians, patients, and families in the decision-making process around outpatient management. These data will inform implementation studies to operationalize more personalized discharge practices for children receiving AML chemotherapy.

## Notes

Supplement.
eMethods.
**eTable 1.** Baseline Demographic, Clinical, and Hospital-Level Characteristics for Outpatient vs Inpatient Management for Course-Specific Study Populations **eTable 2.** Days to Initial Discharge, Frequency of Outpatient Management, and Rate and Timing of Readmission After Discharge to Outpatient Management by Treatment Course **eTable 3.** Course-Specific Induction Mortality for Outpatient vs Inpatient Management **eTable 4.** Comparisons of ICU-Level Care Requirements and Resource Utilization Rates for Outpatient vs Inpatient Neutropenia Management Among the Subpopulation Treated at PHIS-Contributing Institutions **eTable 5.** Demographic, Clinical, and Hospital-Level Characteristics by Outpatient vs Inpatient Management for the Early Discharge–Eligible Patients Whose Caregiver Completed the Prospective Assessments of Patient Health-Related Quality of Life **eTable 6.** Demographic, Clinical, and Hospital-Level Characteristics by Outpatient vs Inpatient Management for the Early Discharge–Eligible Patients Whose Caregivers Completed the Sleep Disturbance Scale to Assess Disorders of Initiating and Maintaining Sleep Domain, and the Pediatric Inventory for Parents-Difficulty Assessment **eTable 7.** Demographic, Clinical, and Hospital-Level Characteristics by Outpatient vs Inpatient Management for the Early Discharge–Eligible Patients Population for Whom Caregivers Completed the Comprehensive Score for Financial Toxicity Assessment of Financial Distress **eTable 8.** Prevalence of Financial Difficulties Experienced by Families of Children Treated for AML by Outpatient vs Inpatient Management **eFigure 1.** Flowchart Depicting Assembly of Final Analytic Study Population From Overall Health Record Abstraction Cohort of Newly Diagnosed Pediatric Patient With Acute Myeloid Leukemia **eFigure 2.** Distribution of Timing of Discharge Relative to the Last Day of Chemotherapy for Each Course **eFigure 3.** Flowchart Depicting the Study Population Completing HRQOL and Each of the Secondary Patient-Centered Outcome Assessments

## References

[1] Feusner JHMD, Hastings CAMD. Infections in children with acute myelogenous leukemia: concepts of management and prevention. J Pediatr Hematol Oncol. 1995;17(3):234-247. doi:10.1097/00043426-199508000-00005 7620922

[2] Riley LC, Hann IM, Wheatley K, Stevens RF; The MCR Childhood Leukaemia Working Party. Treatment-related deaths during induction and first remission of acute myeloid leukaemia in children treated on the Tenth Medical Research Council Acute Myeloid Leukaemia trial (MRC AML10). Br J Haematol. 1999;106(2):436-444. doi:10.1046/j.1365-2141.1999.01550.x 10460604

[3] Lange BJ, Smith FO, Feusner J, . Outcomes in CCG-2961, a children’s oncology group phase 3 trial for untreated pediatric acute myeloid leukemia: a report from the Children’s Oncology Group. Blood. 2008;111(3):1044-1053. doi:10.1182/blood-2007-04-084293 18000167 PMC2214754

[4] Aplenc R, Meshinchi S, Sung L, . Bortezomib with standard chemotherapy for children with acute myeloid leukemia does not improve treatment outcomes: a report from the Children’s Oncology Group. Haematologica. 2020;105(7):1879-1886. doi:10.3324/haematol.2019.220962 32029509 PMC7327649

[5] Gamis AS, Alonzo TA, Meshinchi S, . Gemtuzumab ozogamicin in children and adolescents with de novo acute myeloid leukemia improves event-free survival by reducing relapse risk: results from the randomized phase III Children’s Oncology Group trial AAML0531. J Clin Oncol. 2014;32(27):3021-3032. doi:10.1200/JCO.2014.55.3628 25092781 PMC4162498

[6] Getz KD, Miller TP, Seif AE, . A comparison of resource utilization following chemotherapy for acute myeloid leukemia in children discharged versus children that remain hospitalized during neutropenia. Cancer Med. 2015;4(9):1356-1364. doi:10.1002/cam4.481 26105201 PMC4567020

[7] Lehrnbecher T, Ethier MC, Zaoutis T, . International variations in infection supportive care practices for paediatric patients with acute myeloid leukaemia. Br J Haematol. 2009;147(1):125-128. doi:10.1111/j.1365-2141.2009.07844.x 19663826

[8] Sung L, Aplenc R, Alonzo TA, Gerbing RB, Lehrnbecher T, Gamis AS. Effectiveness of supportive care measures to reduce infections in pediatric AML: a report from the Children’s Oncology Group. Blood. 2013;121(18):3573-3577. doi:10.1182/blood-2013-01-476614 23471307 PMC3643758

[9] Allan DS, Buckstein R, Imrie KR. Outpatient supportive care following chemotherapy for acute myeloblastic leukemia. Leuk Lymphoma. 2001;42(3):339-346. doi:10.3109/10428190109064590 11699398

[10] Eisele L, Günther F, Ebeling P, Nabring J, Dührsen U, Dürig J. Outpatient management of acute myeloid leukemia after intensive consolidation chemotherapy is feasible and reduces hospital treatment costs. Onkologie. 2010;33(12):658-664. doi:10.1159/000322209 21124036

[11] Gillis S, Dann EJ, Rund D. Selective discharge of patients with acute myeloid leukemia during chemotherapy-induced neutropenia. Am J Hematol. 1996;51(1):26-31. doi:10.1002/(SICI)1096-8652(199601)51:1<26::AID-AJH5>3.0.CO;2-9 8571934

[12] Girmenia C, Latagliata R, Tosti S, . Outpatient management of acute promyelocytic leukemia after consolidation chemotherapy. Leukemia. 1999;13(4):514-517. doi:10.1038/sj.leu.2401375 10214855

[13] Naithani R, Kumar R, Mahapatra M, Agrawal N, Mishra P. Early discharge from hospital after consolidation chemotherapy in acute myeloid leukemia in remission: febrile neutropenic episodes and their outcome in a resource poor setting. Haematologica. 2008;93(9):1416-1418. doi:10.3324/haematol.11696 18603560

[14] Savoie ML, Nevil TJ, Song KW, . Shifting to outpatient management of acute myeloid leukemia: a prospective experience. Ann Oncol. 2006;17(5):763-768. doi:10.1093/annonc/mdl011 16497826

[15] Walter RB, Lee SJ, Gardner KM, . Outpatient management following intensive induction chemotherapy for myelodysplastic syndromes and acute myeloid leukemia: a pilot study. Haematologica. 2011;96(6):914-917. doi:10.3324/haematol.2011.040220 21393334 PMC3105654

[16] Inoue S, Khan I, Mushtaq R, Carson D, Saah E, Onwuzurike N. Postinduction supportive care of pediatric acute myelocytic leukemia: should patients be kept in the hospital? Leuk Res Treatment. 2014;2014:592379. doi:10.1155/2014/592379 25349742 PMC4198778

[17] Orme LM, Babl FE, Barnes C, Barnett P, Donath S, Ashley DM. Outpatient versus inpatient IV antibiotic management for pediatric oncology patients with low risk febrile neutropenia: a randomised trial. Pediatr Blood Cancer. 2014;61(8):1427-1433. doi:10.1002/pbc.25012 24604835

[18] Sung L, Feldman BM, Schwamborn G, . Inpatient versus outpatient management of low-risk pediatric febrile neutropenia: measuring parents’ and healthcare professionals’ preferences. J Clin Oncol. 2004;22(19):3922-3929. doi:10.1200/JCO.2004.01.077 15459214

[19] Szymczak JE, Getz KD, Madding R, . Identifying patient- and family-centered outcomes relevant to inpatient versus at-home management of neutropenia in children with acute myeloid leukemia. Pediatr Blood Cancer. 2018;65(4):65. doi:10.1002/pbc.26927 29286570 PMC6857179

[20] Varni JW, Burwinkle TM, Seid M, Skarr D. The PedsQL 4.0 as a pediatric population health measure: feasibility, reliability, and validity. Ambul Pediatr. 2003;3(6):329-341. doi:10.1367/1539-4409(2003)003<0329:TPAAPP>2.0.CO;2 14616041

[21] Streisand R, Braniecki S, Tercyak KP, Kazak AE. Childhood illness-related parenting stress: the pediatric inventory for parents. J Pediatr Psychol. 2001;26(3):155-162. doi:10.1093/jpepsy/26.3.155 11259517

[22] Bruni O, Ottaviano S, Guidetti V, . The Sleep Disturbance Scale for Children (SDSC): construction and validation of an instrument to evaluate sleep disturbances in childhood and adolescence. J Sleep Res. 1996;5(4):251-261. doi:10.1111/j.1365-2869.1996.00251.x 9065877

[23] de Souza JA, Yap BJ, Wroblewski K, . Measuring financial toxicity as a clinically relevant patient-reported outcome: the validation of the Comprehensive Score for Financial Toxicity (COST). Cancer. 2017;123(3):476-484. doi:10.1002/cncr.30369 27716900 PMC5298039

[24] Aplenc R, Fisher BT, Huang YS, . Merging of the National Cancer Institute-funded cooperative oncology group data with an administrative data source to develop a more effective platform for clinical trial analysis and comparative effectiveness research: a report from the Children’s Oncology Group. Pharmacoepidemiol Drug Saf. 2012;21(suppl 2):37-43. doi:10.1002/pds.3241 22552978 PMC3359580

[25] Maude SL, Fitzgerald JC, Fisher BT, . Outcome of pediatric acute myeloid leukemia patients receiving intensive care in the United States. Pediatr Crit Care Med. 2014;15(2):112-120. doi:10.1097/PCC.0000000000000042 24366507 PMC4407366

[26] Bakhshi S, Singh P, Swaroop C. Outpatient consolidation chemotherapy in pediatric acute myeloid leukemia: a retrospective analysis. Hematology. 2009;14(5):255-260. doi:10.1179/102453309X446144 19843379

[27] Linder LA, Christian BJ. Characteristics of the nighttime hospital bedside care environment (sound, light, and temperature) for children with cancer. Cancer Nurs. 2011;34(3):176-184. doi:10.1097/NCC.0b013e3181fc52d0 21522058 PMC3085834

[28] Nagarajan R, Gerbing R, Alonzo T, . Quality of life in pediatric acute myeloid leukemia: Report from the Children’s Oncology Group. Cancer Med. 2019;8(9):4454-4464. doi:10.1002/cam4.2337 31190442 PMC6675729

[29] Getz KD, Li Y, Alonzo TA, . Comparison of in-patient costs for children treated on the AAML0531 clinical trial: a report from the Children’s Oncology Group. Pediatr Blood Cancer. 2015;62(10):1775-1781. doi:10.1002/pbc.25569 25946708 PMC4546551

[30] Amrhein V, Greenland S, McShane B. Scientists rise up against statistical significance. Nature. 2019;567(7748):305-307. doi:10.1038/d41586-019-00857-9 30894741

[31] Lash TL. The harm done to reproducibility by the culture of null hypothesis significance testing. Am J Epidemiol. 2017;186(6):627-635. doi:10.1093/aje/kwx261 28938715

