# Supplementary material for: Medical Outcomes, Quality of Life, and Family Perceptions for Outpatient vs Inpatient Neutropenia Management After Chemotherapy for Pediatric Acute Myeloid Leukemia
Source: JAMA Netw Open. 2021 Oct 28;4(10):e2128385. doi: 10.1001/jamanetworkopen.2021.28385 (PMC8554641; doi:10.1001/jamanetworkopen.2021.28385)
Supplement: Supplement. — eMethods. eTable 1. Baseline Demographic, Clinical, and Hospital-Level Characteristics for Outpatient vs Inpatient Management for Course-Specific Study Populations eTable 2. Days to Initial Discharge, Frequency of Outpatient Management, and Rate and Timing of Readmission After Discharge to Outpatient Management by Treatment Course eTable 3. Course-Specific Induction Mortality for Outpatient vs Inpatient Management eTable 4. Comparisons of ICU-Level Care Requirements and Resource Utilization Rates for Outpatient vs Inpatient Neutropenia Management Among the Subpopulation Treated at PHIS-Contributing Institutions eTable 5. Demographic, Clinical, and Hospital-Level Characteristics by Outpatient vs Inpatient Management for the Early Discharge–Eligible Patients Whose Caregiver Completed the Prospective Assessments of Patient Health-Related Quality of Life eTable 6. Demographic, Clinical, and Hospital-Level Characteristics by Outpatient vs Inpatient Management for the Early Discharge–Eligible Patients Whose Caregivers Completed the Sleep Disturbance Scale to Assess Disorders of Initiating and Maintaining Sleep Domain, and the Pediatric Inventory for Parents-Difficulty Assessment eTable 7. Demographic, Clinical, and Hospital-Level Characteristics by Outpatient vs Inpatient Management for the Early Discharge–Eligible Patients Population for Whom Caregivers Completed the Comprehensive Score for Financial Toxicity Assessment of Financial Distress eTable 8. Prevalence of Financial Difficulties Experienced by Families of Children Treated for AML by Outpatient vs Inpatient Management eFigure 1. Flowchart Depicting Assembly of Final Analytic Study Population From Overall Health Record Abstraction Cohort of Newly Diagnosed Pediatric Patient With Acute Myeloid Leukemia eFigure 2. Distribution of Timing of Discharge Relative to the Last Day of Chemotherapy for Each Course eFigure 3. Flowchart Depicting the Study Population Completing HRQOL and Each of the Secondary Patient-Centered [file jamanetwopen-e2128385-s001.pdf]

Supplemental Online Content

Getz KD, Szymczak JE, Li Y, et al. Medical outcomes, quality of life, and family perceptions for outpatient vs inpatient neutropenia management after chemotherapy for pediatric acute myeloid leukemia. *JAMA Netw Open*. 2021;4(10):e2128385. doi:10.1001/jamanetworkopen.2021.28385

eMethods.

eTable 1. Baseline Demographic, Clinical, and Hospital-Level Characteristics for Outpatient vs Inpatient Management for Course-Specific Study Populations

eTable 2. Days to Initial Discharge, Frequency of Outpatient Management, and Rate and Timing of Readmission After Discharge to Outpatient Management by Treatment Course

eTable 3. Course-Specific Induction Mortality for Outpatient vs Inpatient Management

eTable 4. Comparisons of ICU-Level Care Requirements and Resource Utilization Rates for Outpatient vs Inpatient Neutropenia Management Among the Subpopulation Treated at PHIS-Contributing Institutions

eTable 5. Demographic, Clinical, and Hospital-Level Characteristics by Outpatient vs Inpatient Management for the Early Discharge–Eligible Patients Whose Caregiver Completed the Prospective Assessments of Patient Health-Related Quality of Life

eTable 6. Demographic, Clinical, and Hospital-Level Characteristics by Outpatient vs Inpatient Management for the Early Discharge–Eligible Patients Whose Caregivers Completed the Sleep Disturbance Scale to Assess Disorders of Initiating and Maintaining Sleep Domain, and the Pediatric Inventory for Parents-Difficulty Assessment

eTable 7. Demographic, Clinical, and Hospital-Level Characteristics by Outpatient vs Inpatient Management for the Early Discharge–Eligible Patients Population for Whom Caregivers Completed the Comprehensive Score for Financial Toxicity Assessment of Financial Distress

eTable 8. Prevalence of Financial Difficulties Experienced by Families of Children Treated for AML by Outpatient vs Inpatient Management

eFigure 1. Flowchart Depicting Assembly of Final Analytic Study Population From Overall Health Record Abstraction Cohort of Newly Diagnosed Pediatric Patient With Acute Myeloid Leukemia

eFigure 2. Distribution of Timing of Discharge Relative to the Last Day of Chemotherapy for Each Course

eFigure 3. Flowchart Depicting the Study Population Completing HRQOL and Each of the Secondary Patient-Centered Outcome Assessments

This supplemental material has been provided by the authors to give readers additional information about their work.

© 2021 Getz KD et al. *JAMA Network Open.*

eMethods.

Study Design

A bidirectional observational cohort design with a combination of retrospective and prospective ascertainment of eligible participants was utilized. The source population included newly diagnosed AML patients who received standard intensive frontline chemotherapy at 17 pediatric institutions across the United States between January 2011 and July 2019. Patients were identified by investigators at each institution from their local cancer registries and prospectively at weekly oncology case conferences where all new AML diagnoses are discussed. Patients were ineligible if they had acute promyelocytic leukemia, were older than 18 years at diagnosis, received reduced intensity chemotherapy, or received only a hematopoietic stem cell transplantation at the participating site.

Medical Record Abstraction

Trained abstractors traveled to each participating institution to complete manual medical record abstraction of specific information on demographics, diagnosis, treatment, antimicrobial prophylaxis, inpatient admissions, outpatient clinic and emergency department encounters, and results of blood cultures performed at the treating institution during inpatient admissions and outpatient clinic encounters as well as those submitted from outside institutions. All data were entered directly into electronic case report forms. Study personnel were formally trained using a detailed medical record abstraction guide developed by the study investigators and were required to achieve >95% concordance with their trainer on multiple practice abstractions before commencing formal study chart reviews.

Following each round of chart abstraction, collected data were extracted from the REDCap database and evaluated for quality and completeness using programs designed to identify missing information and potentially erroneous data entries. All generated queries were verified against the patient medical records; the resolution for each interrogation or confirmation of the accuracy of the abstracted data upon verification against the EMR was recorded in the query forms and archived. This standardized approach to chart abstraction ensured comparable quality and consistency in data collection across all participating institutions and across all years of the study.

© 2021 Getz KD et al. *JAMA Network Open.*

Furthermore, this practice resulted in near complete capture of exposure, outcome, and relevant potential confounders of the associations of interest.

Institutional IRB determined that patient/parent consent to medical record abstraction was not required thus allowing for complete capture of the target population for assessment of the primary medical outcomes, occurrence of bacteremia during post-chemotherapy neutropenia and time to the initiation of the next chemotherapy course.

Institutional Survey

Given that the exposure of interest (outpatient versus inpatient management) was determined by institutional practice, there was the potential for confounding by other site-level practices related to management strategy and the outcomes of interest. In an attempt to mitigate such confounding, data on institutional standard practice with respect to infection control (i.e., utilization of systemic antimicrobial prophylaxis, utilization of chlorhexidine gluconate bathing) and central line management (i.e., antibiotic and ethanol catheter lock therapy) were acquired via surveys to site investigators.

Qualitative Interviews

From November 2015 to February 2017, in-depth, semistructured interviews were conducted with children who completed AML chemotherapy at nine of the participating institutions and their families as previously described.[5] The goals of the interviews were to identify patient- and family-centered outcomes related to neutropenia management, to elicit perspectives about the impact of the patient's illness on family life, and their beliefs about the risks and benefits of different neutropenia management strategies.

Audio files were transcribed and uploaded to NVivo 11. Data were analyzed using a modified grounded theory approach in which two analysts systematically identified themes and patterns in the text via open coding. All transcripts were read line-by-line and salient concepts were identified, defined, and refined.

Prospective Questionnaires

Between June 2016 and May 2019 newly diagnosed AML patients at 15 institutions who were receiving standard intensive frontline chemotherapy, able to read English or Spanish if 8 years of age or older, and whose legal guardian of the patient was able to read English or Spanish were eligible to participate in a series of

© 2021 Getz KD et al. *JAMA Network Open.*

questionnaires administered at two time points during a single post-Induction I chemotherapy course: prior to the neutropenia onset (baseline) and following resolution of neutropenia but before the start of their next chemotherapy course (follow-up). Fourteen of the institutions were a subset of the 17 institutions contributing to the assessment of the primary medical outcomes plus one additional institution which did not contribute to that analysis due to an ongoing local study with overlapping objectives.

Ascertainment of potential participants occurred on a weekly basis by either study coordinator attendance at the weekly Leukemia Team Clinical Conference or review of the Leukemia Team Clinical Conference minutes. At sites without a weekly Leukemia Team Clinical Meeting, the study coordinator contacted the attending physician or fellows on the Oncology service to identify new patients. Once identified, study personnel reviewed each patient against the study inclusion and exclusion criteria using an electronic screening tool. Once a patient was determined to meet an exclusion criterion a notification of ineligibility was generated and the reason for ineligibility was saved along with the patient's study ID. Site coordinators approached each eligible patient for consent prior to the end of chemotherapy in the study course and completed the documentation of consent forms in the electronic screening eligibility tool to record the date of consent, or in cases of non-consent the reason for the refusal to participate. Site personnel informed the coordinating center about eligible patients and their consent through bi-weekly updates and regular email correspondence throughout the week. Regular contact between the coordinating center and site personnel at each institution via a combination of bi-weekly email updates and monthly calls with personnel from all institutions allowed for consistent monitoring of eligibility and progress of enrolled participants through the study. To show our appreciation for the patient and caregiver's time to participate, they received $25 gift cards after completing the baseline and follow-up assessments for a total of $50.

The questionnaires included a baseline survey to obtain socioeconomic information not available in the medical record, the acute PedsQL™ 4.0 Generic Core Scales[1], Pediatric Inventory for Parents-Difficulty assessment (PIP-D)[2], the Sleep Disturbance Scale for Children-Disorders of Initiating and Maintaining Sleep domain (SDCS-DIMS)[3], and a modified Comprehensive Score for financial Toxicity (COST)[4]. The PedsQL and COST were administered at baseline and follow-up, whereas the SDSC-DIMS and PIP-D were administered at

© 2021 Getz KD et al. *JAMA Network Open.*

follow-up only. The SDSC-DIMS, PIP-D and COST assessments were incorporated based on the results of the analysis of qualitative interviews[5] and were rolled out as they were developed and the amended IRB protocols were approved by participating sites. Therefore, not every prospective participant who consented to the questionnaires was administered all the assessments.

PedsQL™ 4.0 Generic Core scales use a 7-day retrospective reflection time frame for the assessment of four domains: physical functioning, emotional functioning, social functioning, and school functioning. Respondents document responses to each question using a 5-point Likert scale anchored by never a problem (0) to almost always a problem (4). These scales demonstrate internal reliability acceptable for group comparisons (PedsQL Generic Core Total Scale Score, Cronbach's $\alpha = 0.93$ for parent report) and have been validated to be sensitive to change over time in children with cancer.[1,6,7] The SDSC-DIMS assesses sleep indices such as latency and duration, night awakenings, and reluctance to go to bed. All items are measured on a five-point Likert scale from 1 (never) to 5 (always). The SDSC is reported to have high internal consistency among both healthy (α=0.79) and sleep disordered participants (α=0.71), as well as high test–retest reliability (r=0.71).[3] The PIP-D assesses stress-related difficulty with events faced by parents of children with serious illness across four domains: communication (e.g., with child, partner, or health care team), emotional functioning (e.g., impact of illness on sleeping and mood), child's medical care (e.g., carrying out medical regimen), and role functioning (e.g., impact of illness on parent's ability to work and care for other children). All items are rated on a five-point Likert scale from 1 (not at all difficult) to 5 (extremely difficult). Internal consistency and reliability for the PIP was demonstrated to be high (α= 0.80-0.96) and scores were significantly correlated with a measures of anxiety and parenting stress, demonstrating construct validity.[2] The COST assessment includes 11 statements about the financial situation of the caregiver/family in relation to the child's treatment adapted from existing literature. A 5-point Likert response scale is used for the parent to indicate the degree to which they agree with each statement, 0 (not at all) to 4 (very much). The COST measure has been shown to have internal consistency and test-retest reliability, and scores were found to be correlated with income and psychosocial distress.[4]

Pediatric Health Information System

Pediatric Health Information System (PHIS) data were identified and merged for patients treated at PHIS-contributing institutions. PHIS is an administrative database containing inpatient and emergency department observational database from 49 free-standing children's hospitals. Data include demographics, dates of service, discharge disposition, and daily inpatient billing data for medications, laboratory tests, imaging procedures, clinical services, and supplies. Patients are assigned a unique identifier which allows records to be linked longitudinally across admissions. Data are anonymized at the time of submission and subject to a number of reliability and validity checks before inclusion in the database. Data quality is assured through a joint effort between the Children's Hospital Association and participating hospitals. Methods for merging PHIS administrative data with external data sources have been described previously.[8]

Daily billing data were used to identify patients receiving any intensive care unit-level resources[9] and to determine cumulative rates of utilization (days of use per 1000 inpatient days) for the following specific resources over the follow-up period: antibiotic, antifungal, antiviral and vasopressor medications, parenteral nutrition, blood product replacement and supplemental oxygen.

Exposure

Quantitative analyses were restricted to patients who survived to the completion of course-specific chemotherapy and were determined to be eligible for early discharge to outpatient management. Patients were considered discharge-eligible if there was no evidence of fever (i.e., temperature of 38.4°C or higher), microbiologically-documented infection (i.e., positive blood culture) or intensive care unit (ICU) level requirements (i.e., vasoactive infusions, supplemental oxygen, or dialysis) within 3 days of the last dose of chemotherapy in the given course. Patients discharged within the 3 days after chemotherapy completion were categorized as discharged to outpatient neutropenia management. Patients meeting discharge eligibility criteria but remaining in the hospital more than 3 days after chemotherapy completion were categorized as inpatient management.

Outcomes

The primary medical outcomes were course-specific occurrence of bacteremia during post-chemotherapy neutropenia and time to the initiation of the next chemotherapy course. Follow-up for identification of bacteremia began three days after course-specific chemotherapy completion and continued until the earliest of death, neutrophil count recovery, the start of the next course of chemotherapy or 50 days from the start of chemotherapy in the course. Time to the start of the next chemotherapy course was measured as the difference in days from the first day of systemic chemotherapy in the next course and three days after chemotherapy completion in the preceding course. Secondary outcomes included course-specific mortality, and for the subset of patients treated at hospitals contributing to PHIS receipt of ICU-level care and resource utilization rates.

The primary patient-centered outcome was health-related quality of life (HRQOL) measured using parent-proxy responses to the acute PedsQL™ 4.0 Generic Core Scales. Items were reverse scored and linearly transformed to a scale of 0 to 100 such that higher scores reflect better HRQOL. The Total score was calculated as the sum of all item-specific scores divided by the number of answered items.[1,10] In addition to the Total score, a Physical Health subscore was similarly computed using the physical functioning domain items, and a Psychosocial Health subscore was created using the emotional and social domain items.

Secondary patient-centered outcomes included patient sleep disturbance as measured by the SDCS-DIMS, parental stress as measured by the PIP-D, and financial distress as measured by the modified COST. Responses to each of the items on the PIP-D were summed to obtain a total score reflecting the amount of difficulty experienced when handling events faced by parents of children with serious illness. Responses to each of the items on the SDSC-DIMS subscale and separately for the FTA were summed to get a total score. Higher scores indicate greater difficulty and increased pediatric parenting stress.

Planned Sample Size and Power

We expected to identify a total of 533 patients newly diagnosed with AML across the participating centers. Assuming 90% of identified patients would meet the early-discharge eligibility criteria, the anticipated study population for the primary medical outcomes was 480 patients, of which 27% were expected to be managed as outpatients and the remaining managed as inpatients during neutropenia. Assuming each patient

© 2021 Getz KD et al. *JAMA Network Open.*

contributes at least 2 courses and a within-subject correlation of 0.15, we would have 96.8% power to detect a 15% difference in bacteremia rates between groups. For time to next chemotherapy course, we used a 2-sample *t* test in the power calculation to be conservative. Assuming an average time to next chemotherapy course of 29 days, a standard deviation of 10 days (based on preliminary data) and that each patient would contribute at least 2 courses with a within-subject correlation of 0.15, we would have 98.1% power to detect a 3-day difference.

We expected to identify and consent a total of 120 patients to the prospective assessment of HRQOL over the study period. Assuming 90% of consented patients would meet the early-discharge eligibility criteria, the anticipated analytic study population for assessment of HRQOL was 108 patients. Based on the literature, the expected standard deviation for the PedsQL total score was 20 (on a scale of 0-100) and a minimal clinically important difference in change scores between groups was 7 to 10 points. The power calculation was conducted under the framework of a multivariate general linear hypothesis for general linear models with a significance level of α=0.05 (Wilks λ test). If there were no correlations, we anticipated having 80% power to detect a 15.0-point difference in the mean change scores between the compared groups; if the correlation was as high as 0.8, we anticipated having 80% power to detect a 6.8-point difference in the mean change scores between the two groups.

Statistical Analyses

Histograms of the timing of discharge relative to the completion of chemotherapy were plotted for each course. The frequencies of early discharge and course-specific case fatality rates (n, %) were tabulated by course and patient characteristics and were compared using chi-square tests or Fishers exacts tests. Log-binomial regression was used to estimate risk ratios with 95% confidence intervals (CI) comparing the incidence of bacteremia following outpatient versus inpatient neutropenia management strategy. Before statistical model fitting, the normality of the distribution of times to next chemotherapy course was assessed. Given that the normality assumption was not violated, standard linear regression models were utilized to compare time to next course for outpatient versus inpatient management. All course-specific comparisons employed general estimating equation (GEE) methods to account for non-independence of observations from

© 2021 Getz KD et al. *JAMA Network Open.*

patients from the same institution. We also evaluated the associations collapsed across all treatment courses, summary measures of the associations across courses were computed utilizing GEE methods to account for non-independence between courses contributed by the same patient.

Analysis of covariance was employed to test the association between neutropenia management strategy and the PedsQL™ scores (and COST scores). Linear regression models were used to compare follow-up PIP-D scores, SDSC-DIMS scores, and change in COST scores for outpatient versus inpatient management.

Propensity score analyses were employed to adjust for potential confounding by baseline covariates in analyses. Propensity scores were derived from the predicted probabilities estimated from regression models of the use of outpatient versus inpatient management during neutropenia conditional on all baseline factors determined from bivariate analyses to be true confounders (i.e., those associated with both exposure and outcome with p-value <0.2) and those determined to be potential confounders (i.e., those associated only with the outcome interests). Patients were then stratified into five groups using quintiles of the estimated propensity score. Balance of covariates between outpatient and inpatient management groups was assessed before and after application of the generated propensity score. Control for confounding was accomplished through adjustment for the propensity score quintiles as well as any remaining unbalanced patient- or hospital-level confounders.

REFERENCES

1. Varni JW, Burwinkle TM, Seid M, Skarr D. The PedsQL™* 4.0 as a Pediatric Population Health Measure: Feasibility, Reliability, and Validity. *Ambulatory Pediatrics.* 2003;3(6):329-341.
2. Streisand R, Braniecki S, Tercyak KP, Kazak AE. Childhood illness-related parenting stress: the pediatric inventory for parents. *J Pediatr Psychol.* 2001;26(3):155-162.
3. Bruni O, Ottaviano S, Guidetti V, et al. The Sleep Disturbance Scale for Children (SDSC). Construction and validation of an instrument to evaluate sleep disturbances in childhood and adolescence. *J Sleep Res.* 1996;5(4):251-261.

© 2021 Getz KD et al. *JAMA Network Open.*

4. de Souza JA, Yap BJ, Wroblewski K, et al. Measuring financial toxicity as a clinically relevant patient-reported outcome: The validation of the COmprehensive Score for financial Toxicity (COST). *Cancer.* 2017;123(3):476-484.

5. Szymczak JE, Getz KD, Madding R, et al. Identifying patient- and family-centered outcomes relevant to inpatient versus at-home management of neutropenia in children with acute myeloid leukemia. *Pediatr Blood Cancer.* 2018;65(4).

6. Varni JW, Burwinkle TM, Katz ER, Meeske K, Dickinson P. The PedsQL in pediatric cancer: reliability and validity of the Pediatric Quality of Life Inventory Generic Core Scales, Multidimensional Fatigue Scale, and Cancer Module. *Cancer.* 2002;94(7):2090-2106.

7. Klaassen RJ, Krahn M, Gaboury I, et al. Evaluating the ability to detect change of health-related quality of life in children with Hodgkin disease. *Cancer.* 2010;116(6):1608-1614.

8. Aplenc R, Fisher BT, Huang YS, et al. Merging of the National Cancer Institute-funded cooperative oncology group data with an administrative data source to develop a more effective platform for clinical trial analysis and comparative effectiveness research: a report from the Children's Oncology Group. *Pharmacoepidemiol Drug Saf.* 2012;21 Suppl 2:37-43.

9. Maude SL, Fitzgerald JC, Fisher BT, et al. Outcome of pediatric acute myeloid leukemia patients receiving intensive care in the United States. *Pediatr Crit Care Med.* 2014;15(2):112-120.

10. Varni JW. Scaling and Scoring of the Pediatric Quality of Life Inventory, PedsQL. *MAPI Research Trust.* 2008.

© 2021 Getz KD et al. *JAMA Network Open.*

eTable 1. Baseline Demographic, Clinical, and Hospital-Level Characteristics for Outpatient vs Inpatient Management for Course-Specific Study Populations

	Induction II			Intensification I			Intensification II			Intensification III		
	Outpatient	Inpatient	p-value	Outpatient	Inpatient	p-value	Outpatient	Inpatient	p-value	Outpatient	Inpatient	p-value
Total N	114	379		104	270		56	229		7	37	
**Demographics**												
Gender			0.435			0.838			0.930			1.000
Female	53 (46.5)	192 (50.7)		50 (48.1)	133 (49.3)		28 (50.0)	116 (50.7)		4 (57.1)	20 (54.1)	
Male	61 (53.5)	187 (49.3)		54 (51.9)	137 (50.7)		28 (50.0)	113 (49.3)		3 (42.9)	17 (45.9)	
Age at Diagnosis			<0.001			<0.001			<0.001			0.442
0 to 1 years	18 (15.8)	145 (38.3)		18 (17.3)	111 (41.1)		5 (8.9)	93 (40.6)		1 (14.3)	13 (35.1)	
2 to 10 years	45 (39.5)	107 (28.2)		33 (31.7)	70 (25.9)		21 (37.5)	64 (28.0)		3 (42.9)	9 (24.3)	
11+ years	51 (44.7)	127 (33.5)		53 (51.0)	89 (33.0)		30 (53.6)	72 (31.4)		3 (42.9)	15 (40.5)	
Race			<0.001			0.016			0.359			0.369
White	52 (45.6)	224 (59.1)		53 (51.0)	155 (57.4)		26 (46.4)	134 (58.5)		3 (42.9)	24 (64.9)	
Black	17 (14.9)	74 (19.5)		13 (12.5)	52 (19.3)		11 (19.6)	40 (17.5)		2 (28.6)	4 (10.8)	
Asian	11 (9.7)	26 (6.9)		8 (7.7)	22 (8.2)		4 (7.1)	13 (5.7)		1 (14.3)	1 (2.7)	
Other	33 (29.0)	44 (11.6)		28 (26.9)	33 (12.2)		14 (25.0)	35 (15.3)		1 (14.3)	5 (13.5)	
Not recorded in EMR	1 (0.9)	11 (2.9)		2 (1.9)	8 (3.0)		1 (1.8)	7 (3.1)		0 (0.0)	3 (8.1)	
Hispanic Ethnicity	29 (25.4)	77 (20.3)	0.243	24 (23.1)	59 (21.9)	0.798	16 (28.6)	50 (21.8)	0.284	1 (14.3)	7 (18.9)	1.00
Insurance at course start			0.035			0.437			0.259			
Any Private	46 (40.4)	181 (47.8)		48 (46.2)	125 (46.3)		22 (39.3)	106 (46.3)		3 (42.9)	13 (35.1)	
Public only or Uninsured	59 (51.8)	174 (45.9)		50 (48.1)	132 (48.9)		34 (60.7)	111 (48.5)		4 (57.1)	22 (59.5)	
Other	4 (3.5)	21 (5.5)		3 (2.9)	11 (4.1)		0 (0)	9 (3.2)		0 (0)	1 (2.7)	
Not recorded in EMR	5 (4.4)	3 (0.80)		3 (2.9)	2 (0.7)		0 (0)	3 (1.3)		0 (0)	1 (2.7)	
**Clinical Characteristics**												
Year of Diagnosis			0.192			0.986			0.152			0.058
2011-2013	34 (29.8)	100 (26.4)		30 (28.9)	78 (28.9)		23 (41.1)	67 (29.3)		3 (42.9)	4 (10.8)	
2014-2016	54 (47.4)	159 (42.0)		44 (42.3)	112 (41.5)		22 (39.3)	93 (40.6)		1 (14.3)	3 (8.1)	
2017-2019	26 (22.8)	120 (31.7)		30 (28.9)	80 (29.6)		11 (19.6)	69 (30.1)		3 (42.9)	30 (81.1)	
AML Diagnosis Type			0.473			0.402			0.355			
De novo	110 (96.5)	358 (94.5)		101 (97.1)	266 (98.5)		55 (98.2)	228 (99.6)		7 (100)	37 (100)	
Secondary or from TMD	4 (3.5)	21 (5.5)		3 (2.9)	4 (1.5)		1 (1.8)	1 (0.4)		0 (0)	0 (0)	
Risk Classification			0.031			0.293			0.500			0.671
Low	83 (72.8)	253 (66.8)		69 (66.4)	177 (65.6)		47 (83.9)	201 (87.8)		6 (85.7)	32 (86.5)	
Intermediate	10 (8.8)	17 (4.5)		9 (8.7)	13 (4.8)		5 (8.9)	11 (4.8)		1 (14.3)	2 (5.4)	
High	21 (18.4)	109 (28.8)		26 (25.0)	80 (29.6)		4 (7.1)	17 (7.4)		0 (0)	3 (8.1)	

© 2021 Getz KD et al. *JAMA Network Open.*

eTable 1 (continued).

	Induction II			Intensification I			Intensification II			Intensification III		
	Outpatient	Inpatient	p-value	Outpatient	Inpatient	p-value	Outpatient	Inpatient	p-value	Outpatient	Inpatient	p-value
Trial Enrollment			0.002			0.039			0.168			1.00
No	64 (56.1)	238 (62.8)		64 (61.5)	176 (65.2)		30 (53.6)	147 (64.2)		6 (85.7)	35 (94.6)	
Yes - COG Trial	32 (28.1)	120 (31.7)		25 (24.0)	77 (28.5)		19 (33.9)	68 (29.7)		1 (14.3)	2 (5.4)	
Yes - St. Jude Trial	18 (15.8)	21 (5.5)		15 (14.4)	17 (6.3)		7 (12.5)	14 (6.1)		0 (0)	0 (0)	
Chemotherapy Regimen			0.002			0.069			0.909			0.692
ADE	95 (83.3)	246 (64.9)		0 (0)	2 (0.7)		0 (0)	0 (0)		0 (0)	0 (0)	
AE	0 (0)	6 (1.6)		83 (79.8)	212 (78.5)		2 (3.6)	6 (2.6)		1 (14.3)	3 (8.1)	
MA	13 (11.4)	98 (25.9)		18 (17.3)	28 (10.4)		34 (60.7)	146 (63.8)		0 (0)	4 (10.8)	
HD AraC	2 (1.8)	5 (1.3)		1 (1.0)	11 (4.1)		17 (30.4)	64 (28.0)		6 (85.7)	28 (75.7)	
Other regimen	4 (3.5)	4 (3.5)		2 (1.9)	17 (6.3)		3 (5.4)	13 (5.7)		0 (0)	2 (5.4)	
Central line type at start of course			0.423			0.975			0.640			0.312
Tunneled catheter	76 (66.7)	257 (67.8)		74 (71.2)	189 (70.0)		35 (62.5)	155 (67.7)		4 (57.1)	27 (73.0)	
Implanted port	8 (7.0)	30 (7.9)		8 (7.7)	22 (8.2)		7 (12.5)	22 (9.6)		1 (14.3)	1 (14.3)	
PICC	29 (25.4)	92 (24.3)		22 (21.2)	59 (21.9)		14 (25.0)	52 (22.7)		2 (28.6)	2 (28.6)	
No central line	1(0.9)	0 (0)		0	0		0	0		0	0	
Any PJP Coverage	100 (87.7)	369 (97.4)	<0.001	91 (87.5)	260 (96.3)	0.002	46 (82.1)	221 (96.5)	<0.001	6 (85.7)	34 (91.9)	0.514
Any Antibacterial prophylaxis	43 (37.7)	166 (43.8)	0.280	44 (42.3)	120 (44.4)	0.729	27 (48.2)	98 (42.8)	0.548	4 (57.1)	24 (64.9)	0.692
Broad Gram positive coverage	41 (36.0)	163 (43.0)	0.194	40 (38.5)	117 (43.3)	0.415	26 (46.4)	96 (41.9)	0.550	3 (42.9)	24 (64.9)	0.402
Broad Gram negative coverage	41 (36.0)	161 (42.5)	0.233	39 (37.5)	116 (43.0)	0.351	25 (44.6)	89 (38.9)	0.449	3 (42.9)	21 (56.8)	0.684
Antipseudomonal coverage	40 (35.1)	159 (42.0)	0.231	35 (33.7)	115 (42.6)	0.127	24 (44.6)	86 (37.6)	0.361	3 (42.9)	19 (51.4)	0.681
Broad Anaerobic coverage	8 (7.0)	13 (3.4)	0.112	2 (1.92)	16 (5.9)	0.174	4 (7.1)	13 (5.7)	0.752	0 (0)	4 (10.8)	0.362
MRSA coverage	31 (27.2)	120 (31.7)	0.364	25 (24.0)	85 (31.5)	0.159	21 (37.5)	73 (31.9)	0.423	3 (42.9)	16 (43.2)	1.00
**Hospital Anti-infective Practices**												
Systemic antibacterial prophylaxis	47 (41.2)	170 (44.9)	0.494	48 (46.2)	119 (44.1)	0.717	13 (23.2)	107 (46.7)	0.001	1 (14.3)	16 (43.2)	0.220
Line lock therapy	23 (20.2)	113 (29.8)	0.044	17 (16.4)	83 (30.7)	0.005	17 (30.4)	55 (24.0)	0.328	2 (28.6)	7 (18.9)	0.619
Antiseptic bathing protocol	79 (69.3)	237 (62.5)	0.187	78 (75.0)	167 (61.9)	0.017	31 (55.4)	153 (66.8)	0.108	5 (71.4)	19 (51.4)	0.428

AML= acute myelpid leukemia, COG=Children's Oncology Group, TMD= Transient myeloproliferative disorder, EMR= electronic medical record, PICC= Peripherally inserted central catheter, PJP= Pneumocystis jiroveci pneumonia , MRSA= methicillin-resistant Staphylococcus aureus

Other ace category includes American Indian/Alaska Native, Native Hawaiian/Pacific Islander, and those recorded as "Other race"

© 2021 Getz KD et al. *JAMA Network Open.*

eTable 2. Days to Initial Discharge, Frequency of Outpatient Management, and Rate and Timing of Readmission After Discharge to Outpatient Management by Treatment Course

		Induction II (N=493)	Intensification I (N=374)	Intensification II (N=285)	Intensification III (N=44)
Discharged during course, N (%)		461 (93.5)	352 (94.1)	278 (97.5)	43 (97.7)
Days to initial discharge, median (IQR)		17 (5-22)	18 (1-23)	24 (17-30)	22 (19-29)
Outpatient management		114 (23.1)	104 (27.8)	56 (19.7)	7 (15.9)
	Readmission, n (%)	89 (78.1)	81 (77.9)	41 (73.2)	6 (85.7)
	Days to initial readmission	8 (5-11)	9 (6-12)	8 (6-9)	6.5 (6-7)
IQR= interquartile range					

© 2021 Getz KD et al. *JAMA Network Open.*

eTable 3. Course-Specific Induction Mortality for Outpatient vs Inpatient Management

Course	Outpatient	Inpatient	Fisher’ s p-value
Induction II	0 (0%)	5 (1.3%)	0.594
Intensification I	1 (0.9%)	0 (0%)	0.278
Intensification II	3 (5.4%)	1 (0.5%)	0.025

© 2021 Getz KD et al. *JAMA Network Open.*

eTable 4. Comparisons of ICU-Level Care Requirements and Resource Utilization Rates for Outpatient vs Inpatient Neutropenia Management Among the Subpopulation Treated at PHIS-Contributing Institutions

	Induction II			Intensification I			Intensification II		
	Discharge Status			Discharge Status			Discharge Status		
	Outpatient	Inpatient	aIRR (95% CI)	Outpatient	Inpatient	aIRR (95% CI)	Outpatient	Inpatient	aIRR (95% CI)
Total N	88	350		77	258		55	197	
Any ICU-Level Care, n (%)	4 (4.6)	9 (2.6)	2.16 (0.48, 9.62)	10 (13.0)	20 (7.8)	2.50 (1.03, 6.06)	6 (10.9)	20 (10.2)	0.80 (0.32, 1.99)
Resource Utilization (days of use per 1000 inpatient days)									
Antibiotics	1352	961	1.37 (1.21, 1.55)*	1053	977	1.03 (0.89, 1.20)	1318	1156	1.14 (1.00, 1.30)*
Antifungals	874	891	0.96 (0.87, 1.04)	895	885	1.01 (0.92, 1.10)	907	954	0.97 (0.87, 1.08)
Antivirals	175	96	1.83 (0.91, 3.71)	266	130	1.49 (0.88, 2.55)	245	167	1.29 (0.60, 2.76)
Vasopressors	7.2	11.4	0.90 (0.24, 3.43)	69.9	22.9	1.98 (1.09, 3.61)*	15.4	21.6	0.65 (0.21, 1.96)
Blood Products	189	124	1.50 (1.10, 2.05)*	162	112	1.49 (1.10, 2.02)*	226	176	1.42 (1.03, 1.95)*
Parenteral Nutrition	71.5	80.9	0.42 (0.15, 1.19)	69.9	84.6	0.66 (0.28, 1.61)	51.7	130	0.27 (0.11, 0.68)
Oxygen Therapy	14.3	11.1	0.45 (0.07, 2.78)	50.7	15.1	6.56 (3.00, 14.3)*	38.0	30.1	1.36 (0.44, 4.23)

*adjusted for propensity score quintile and hospital-level covariates for antibacterial prophylaxis, line lock therapy, antibiotic bathing as standard of care. The propensity score model included age, race, insurance, risk classification, clinical trial enrollment, chemotherapy regimen, PJP coverage, broad gram positive coverage, broad gram negative coverage.

© 2021 Getz KD et al. *JAMA Network Open.*

eTable 5. Demographic, Clinical, and Hospital-Level Characteristics by Outpatient vs Inpatient Management for the Early Discharge–Eligible Patients Whose Caregiver Completed the Prospective Assessments of Patient Health-Related Quality of Life

	Outpatient	Inpatient	p-value
**Demographics**			
Gender			0.237
Female	8 (36.4)	38 (50.7)	
Male	14 (63.6)	37 (49.3)	
Age at Diagnosis			0.059
0 to 1 years	3 (13.6	29 (38.7)	
2 to 10 years	9 (40.9)	17 (22.7)	
11+ years	10 (45.5)	29 (38.7)	
Race			0.037
White	10 (45.5)	56 (74.7)	
Black	5 (22.7)	6 (8.0)	
Asian	2 (9.1)	5 (6.7)	
Other	5 (22.7)	6 (8.0)	
Not recorded in EMR	0 (0)	2 (2.7)	
Hispanic Ethnicity	4 (18.2)	13 (17.3)	0.599
Insurance at course start			0.119
Any Private	7 (31.8)	34 (45.3)	
Public only or Uninsured	13 (59.1)	40 (53.3)	
Other	2 (9.1)	1 (1.3)	
**Clinical Characteristics**			
Risk Classification			0.775
Low	14 (63.6)	50 (66.7)	
Intermediate	2 (9.1)	4 (5.3)	
High	6 (27.3)	21 (28.0)	
Trial Enrollment			<0.001
No	16 (72.7)	74 (98.7)	
Yes - COG Trial	2 (9.1)	1 (1.3)	
Yes - St. Jude Trial	4 (18.2)	0 (0)	
Chemotherapy Regimen			0.061
ADE	12 (54.6)	37 (49.3)	
AE	2 (9.1)	13 (17.3)	
MA	3 (13.6)	11 (14.7)	
HD AraC	0 (0)	10 (13.3)	
Other regimen	5 (22.7)	4 (5.3)	
Central line type at start of course			0.298
Tunneled catheter	17 (77.3)	53 (70.7)	
Implanted port	0 (0)	0 (0)	
PICC	4 (18.2)	4 (18.2)	
No central line	1 (4.6)	1 (4.6)	
Any PCP Coverage	21 (95.5)	72 (96.0)	0.91
Any Antibacterial prophylaxis	13 (59.1)	43 (57.3)	0.883
Broad gram positive coverage	13 (59.1)	42 (56.0)	0.797
Broad gram negative coverage	13 (59.1)	42 (56.0)	0.797
Antipseudomonal coverage	13 (59.1)	41 (54.7)	0.809
MRSA coverage	13 (59.1)	33 (44.0)	0.213

© 2021 Getz KD et al. *JAMA Network Open.*

eTable 5 (continued).

	Outpatient	Inpatient	p-value
**Hospital Characteristics**			
Any antibactierial prophylaxis	12 (54.6)	34 (45.3)	0.447
Line lock therapy	2 (22.7)	16 (21.3)	0.889
Antibiotic bathing	11 (50.0)	43 (57.3)	0.543
**Caregiver Characteristics**			
Annual Household Income			0.787
<$25,000	7 (31.8)	14 (18.7)	
$25,000-34,999	3 (13.6)	10 (13.3)	
$35,000-49,999	3 (13.6)	6 (8.0)	
$50,000-74,999	2 (9.1)	9 (12.0)	
$75,000-99,999	2 (9.1)	5 (6.7)	
$100,000-149,999	3 (13.6)	18 (24.0)	
$150,000+	2 (9.1)	12 (16.0)	
Employment, hours/week			0.360
35+ hours	10 (45.5)	37 (49.3)	
<35 hours	3 (13.6)	7 (9.3)	
Unemployed	8 (36.4)	31 (41.3)	
Educational Attainment			0.048
High school or less	11 (50.0)	17 (22.7)	
Some college or Associate degree	6 (27.3)	20 (26.7)	
Bachelor degree	2 (9.1)	24 (32.0)	
Graduate /Professional degree	3 (13.6)	14 (18.7)	

© 2021 Getz KD et al. *JAMA Network Open.*

eTable 6. Demographic, Clinical, and Hospital-Level Characteristics by Outpatient vs Inpatient Management for the Early Discharge–Eligible Patients Whose Caregivers Completed the Sleep Disturbance Scale to Assess Disorders of Initiating and Maintaining Sleep Domain, and the Pediatric Inventory for Parents-Difficulty Assessment

	Outpatient	Inpatient	p-value
**Patient Demographics (abstracted)**			
Gender			0.321
Female	8 (36.36%)	32 (51.61%)	
Male	14 (63.64%)	30 (48.39%)	
Age at Diagnosis			0.060
0 to 1 years	3 (13.64%)	23 (37.1%)	
2 to 10 years	9 (40.91%)	13 (20.97%)	
11+ years	10 (45.45%)	26 (41.94%)	
Race			0.143
White	11 (50%)	45 (72.58%)	
Black	4 (18.18%)	6 (9.68%)	
Other	7 (31.82%)	11 (17.74%)	
Hispanic Ethnicity	4 (18.18%)	10 (16.13%)	0.538
Insurance at course start			0.316
Any Private	7 (31.82%)	29 (46.77%)	
Public only or Uninsured	15 (68.18%)	33 (53.23%)	
Patient Education Level			0.848
Less than elementary	10 (45.45%)	26 (41.94%)	
Elementary	1 (4.55%)	5 (8.06%)	
Middle School	3 (13.64%)	13 (20.97%)	
High School	8 (36.36%)	18 (29.03%)	
**Clinical Characteristics**			
AML Diagnosis Type			1.000
De novo	22 (100%)	59 (95.16%)	
Secondary or from TMD	0 (0)	3 (4.84)	
Contributed Chemotherapy Course			0.337
Induction II	19 (86.36%)	43 (69.35%)	
Intesification I	3 (13.64%)	9 (14.52%)	
Intensification II	0 (0%)	6 (9.68%)	
Intensification III	0 (0%)	4 (6.45%)	
Risk Classification			0.696
Low	10 (55.56%)	20 (48.78%)	
Intermediate	2 (11.11%)	3 (7.32%)	
High	6 (33.33%)	18 (43.9%)	
Trial Enrollment			<0.001
No	14 (63.64%)	60 (96.77%)	
Yes - COG Trial	3 (13.64%)	2 (3.23%)	
Yes - St. Jude Trial	5 (22.73%)	0 (0%)	
Chemotherapy Regimen			1.000
ADE	11 (50%)	31 (50%)	
AE	3 (13.64%)	9 (14.52%)	
MA	3 (13.64%)	10 (16.13%)	
Other regimen	5 (22.73%)	12 (19.35%)	

© 2021 Getz KD et al. *JAMA Network Open.*

eTable S6 (continued).

	Outpatient	Inpatient	p-value
Central line type at start of course			1.000
Tunneled catheter	17 (77.27%)	46 (74.19%)	
Other line	5 (22.73%)	16 (25.81%)	
Any Antibacterial prophylaxis	21 (95.45%)	61 (98.39%)	0.458
**<u>Caregiver Characteristics</u>**			
Caregiver race			0.389
White	13 (59.09%)	42 (67.74%)	
Black	4 (18.18%)	5 (8.06%)	
Other	5 (22.73%)	15 (24.19%)	
Caregiver Hispanic Ethnicity	5 (23.81%)	12 (19.35%)	0.756
Caregiver Education Level			0.124
High school graduate or less	10 (45.45%)	15 (24.19%)	
Some college, less than Bachelor	6 (27.27%)	17 (27.42%)	
Bachelor+25:38 degree or higher	6 (27.27%)	30 (48.39%)	
Employment - wotk hours per week			0.513
35+hr/wk	9 (42.86%)	32 (51.61%)	
<35hr/wk	3 (14.29%)	4 (6.45%)	
Unemployed	9 (42.86%)	26 (41.94%)	
Household Income			0.418
$100000+/yr	5 (22.73%)	25 (40.98%)	
$50000-$99999/yr	4 (18.18%)	10 (16.39%)	
$25000-$49999/yr	6 (27.27%)	14 (22.95%)	
<$25000/yr	7 (31.82%)	12 (19.67%)	
Number of Adults			0.597
1	3 (15%)	8 (13.33%)	
2	13 (65%)	45 (75%)	
3 or more	4 (20%)	7 (11.67%)	
Number of Children			0.028
1	9 (40.91%)	16 (25.81%)	
2 or 3	8 (36.36%)	41 (66.13%)	0.028
4 or more	5 (22.73%)	5 (8.06%)	
**<u>Hospital Characteristics</u>**			
Any antibactierial prophylaxis			<0.001
None	10 (45.45%)	30 (48.39%)	
Neutropenia	0 (0%)	20 (32.26%)	
Other	12 (54.55%)	12 (19.35%)	
Any antifungal prophylaxis			0.238
None	0 (0%)	5 (8.06%)	
Neutropenia	14 (63.64%)	44 (70.97%)	
Other	8 (36.36%)	13 (20.97%)	
Line lock therapy	5 (22.73%)	15 (24.19%)	1.000
Antibiotic bathing	12 (54.55%)	32 (51.61%)	1.000

© 2021 Getz KD et al. *JAMA Network Open.*

eTable 7. Demographic, Clinical, and Hospital-Level Characteristics by Outpatient vs Inpatient Management for the Early Discharge–Eligible Patients Population for Whom Caregivers Completed the Comprehensive Score for Financial Toxicity Assessment of Financial Distress

**Patient Demographics**			
Gender			0.130
Female	2 (25%)	23 (57.5%)	
Male	6 (75%)	17 (42.5%)	
Age at Diagnosis			0.062
0 to 1 years	0 (0%)	14 (35%)	
2 to 10 years	4 (50%)	8 (20%)	
11+ years	4 (50%)	18 (45%)	
Race			0.251
White	5 (62.5%)	33 (82.5%)	
Black	1 (12.5%)	3 (7.5%)	
Other	2 (25%)	4 (10%)	
Hispanic Ethnicity	1 (12.5%)	5 (12.5%)	1.000
Insurance at course start			1.000
Any Private	3 (37.5%)	18 (45%)	
Public only or Uninsured	5 (62.5%)	22 (55%)	
Patient Education Level			0.581
Less than elementary	2 (25%)	17 (42.5%)	
Elementary	1 (12.5%)	4 (10%)	
Middle School	2 (25%)	12 (30%)	
High School	3 (37.5%)	7 (17.5%)	
**Clinical Characteristics**			
AML Diagnosis Type			1.000
De novo	8 (100%)	40 (100%)	
Secondary or from TMD	()	()	
Contributed Chemotherapy Course			1.000
Induction II	7 (87.5%)	28 (70%)	
Intesification I	1 (12.5%)	6 (15%)	
Intensification II	0 (0%)	4 (10%)	
Intensification III	0 (0%)	2 (5%)	
Risk Classification			0.781
Low	4 (66.67%)	11 (45.83%)	
Intermediate	0 (0%)	2 (8.33%)	
High	2 (33.33%)	11 (45.83%)	
Trial Enrollment			0.068
No	6 (75%)	39 (97.5%)	
Yes - COG Trial	1 (12.5%)	1 (2.5%)	
Yes - St. Jude Trial	1 (12.5%)	0 (0%)	
Chemotherapy Regimen			0.669
ADE	4 (50%)	24 (60%)	
AE	1 (12.5%)	8 (20%)	
MA	2 (25%)	4 (10%)	
Other regimen	1 (12.5%)	4 (10%)	

© 2021 Getz KD et al. *JAMA Network Open.*

eTable 7 (continued).

	Outpatient	Inpatient	p-value
Central line type at start of course			0.418
Tunneled catheter	7 (87.5%)	28 (70%)	
Other line	1 (12.5%)	12 (30%)	
Any Antibacterial prophylaxis	7 (87.5%)	40 (100%)	
**Caregiver Characteristics**			
Caregiver race			0.840
White	6 (75%)	29 (72.5%)	
Black	1 (12.5%)	3 (7.5%)	
Other	1 (12.5%)	8 (20%)	
Caregiver Hispanic Ethnicity	1 (12.5%)	5 (12.5%)	1.000
Caregiver Education Level			0.321
High school graduate or less	4 (50%)	10 (25%)	
Some college, less than bachelor	2 (25%)	10 (25%)	
Bachelor's degree or higher	2 (25%)	20 (50%)	
Employment - wotk hours per week			0.703
35+hr/wk	3 (42.86%)	20 (50%)	
<35hr/wk	1 (14.29%)	3 (7.5%)	
Unemployed	3 (42.86%)	17 (42.5%)	
Household Income			0.559
$100000+/yr	1 (12.5%)	15 (37.5%)	
$50000-$99999/yr	2 (25%)	7 (17.5%)	
$25000-$49999/yr	3 (37.5%)	9 (22.5%)	
<$25000/yr	2 (25%)	9 (22.5%)	
Number of Adults			0.293
1	0 (0%)	7 (17.95%)	
2	6 (85.71%)	30 (76.92%)	
3 or more	1 (14.29%)	2 (5.13%)	
Number of Children			0.288
1	3 (37.5%)	12 (30%)	
2 or 3	3 (37.5%)	24 (60%)	
4 or more	2 (25%)	4 (10%)	
**Hospital Characteristics**			
Antibactierial prophylaxis			0.160
None	5 (62.5%)	20 (50%)	
During neutropenia	0 (0%)	12 (30%)	
Other	3 (37.5%)	8 (20%)	
Line lock therapy	4 (50%)	10 (25%)	0.208
Antibiotic bathing	3 (37.5%)	18 (45%)	1.000

© 2021 Getz KD et al. *JAMA Network Open.*

eTable 8. Prevalence of Financial Difficulties Experienced by Families of Children Treated for AML by Outpatient vs Inpatient Management

		Outpatient, n (%)	Inpatient, n (%)	p-value
Any Notable Financial Problems				0.452
	No	1 (4.5)	1 (1.6)	
	Yes	21 (95.5)	61 (98.4)	
Reported Financial Problems				
	Buying food	3 (13.6)	14 (22.2)	0.540
	Automobile costs (upkeep, gas, insurance)	7 (31.8)	18 (28.6)	0.790
	Paying rent/mortgage	13 (59.1)	16 (25.4)	0.008
	Paying utilities (phone, heat, electric)	8 (36.4)	16 (25.4)	0.410
	Saving for the future	9 (40.9)	25 (39.7)	1.000
Note: the survey on money problems was implemented at the time of the PIP-D and SDSC-DIMS so was available for the broader subpopulation, n=22 outpatients and n=62 inpatients				

© 2021 Getz KD et al. *JAMA Network Open.*

eFigure 1. Flowchart Depicting Assembly of Final Analytic Study Population From Overall Health Record Abstraction Cohort of Newly Diagnosed Pediatric Patient With Acute Myeloid Leukemia

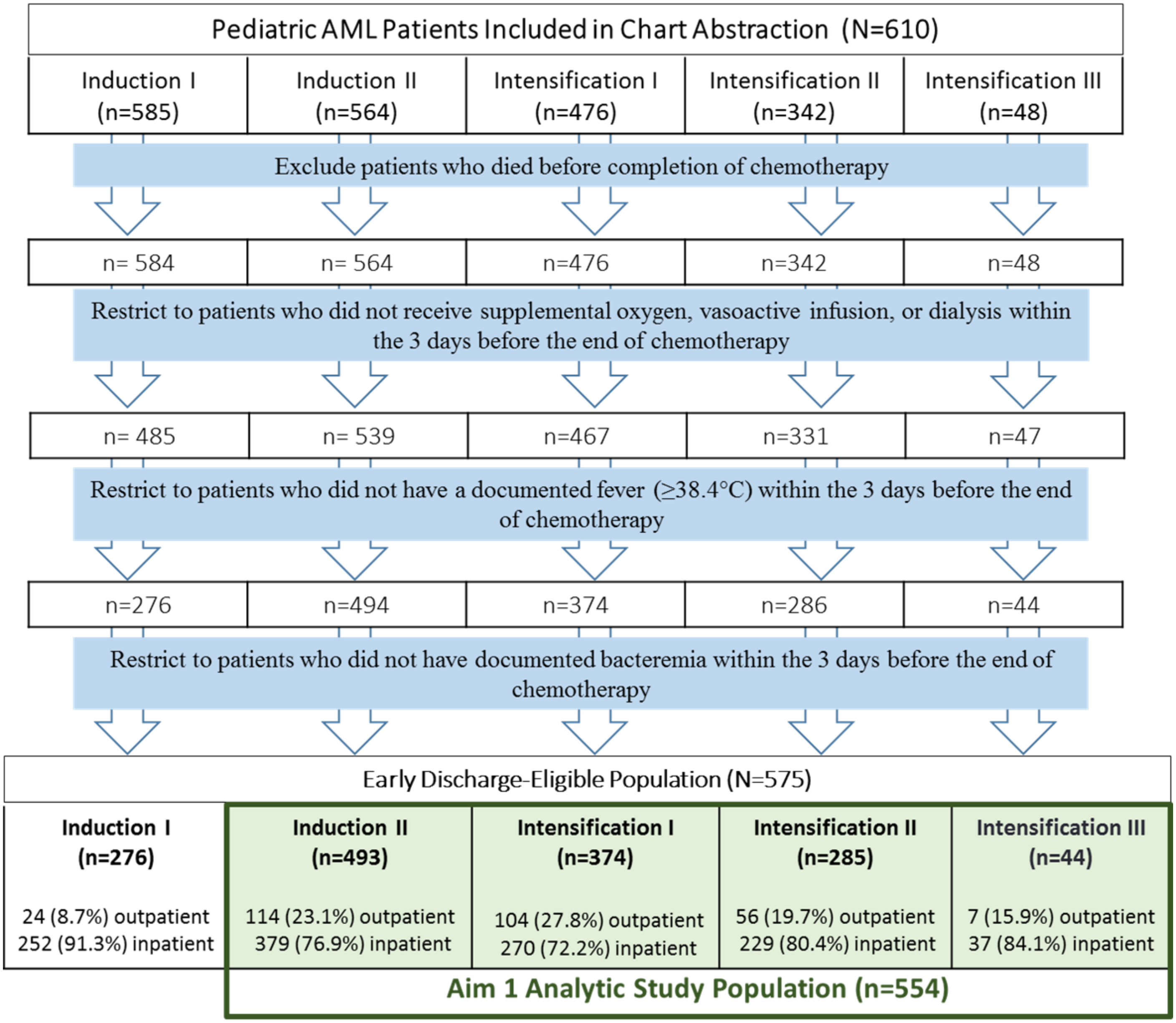

Abbreviations: AML=acute myeloid leukemia

© 2021 Getz KD et al. *JAMA Network Open.*

eFigure 2. Distribution of Timing of Discharge Relative to the Last Day of Chemotherapy for Each Course

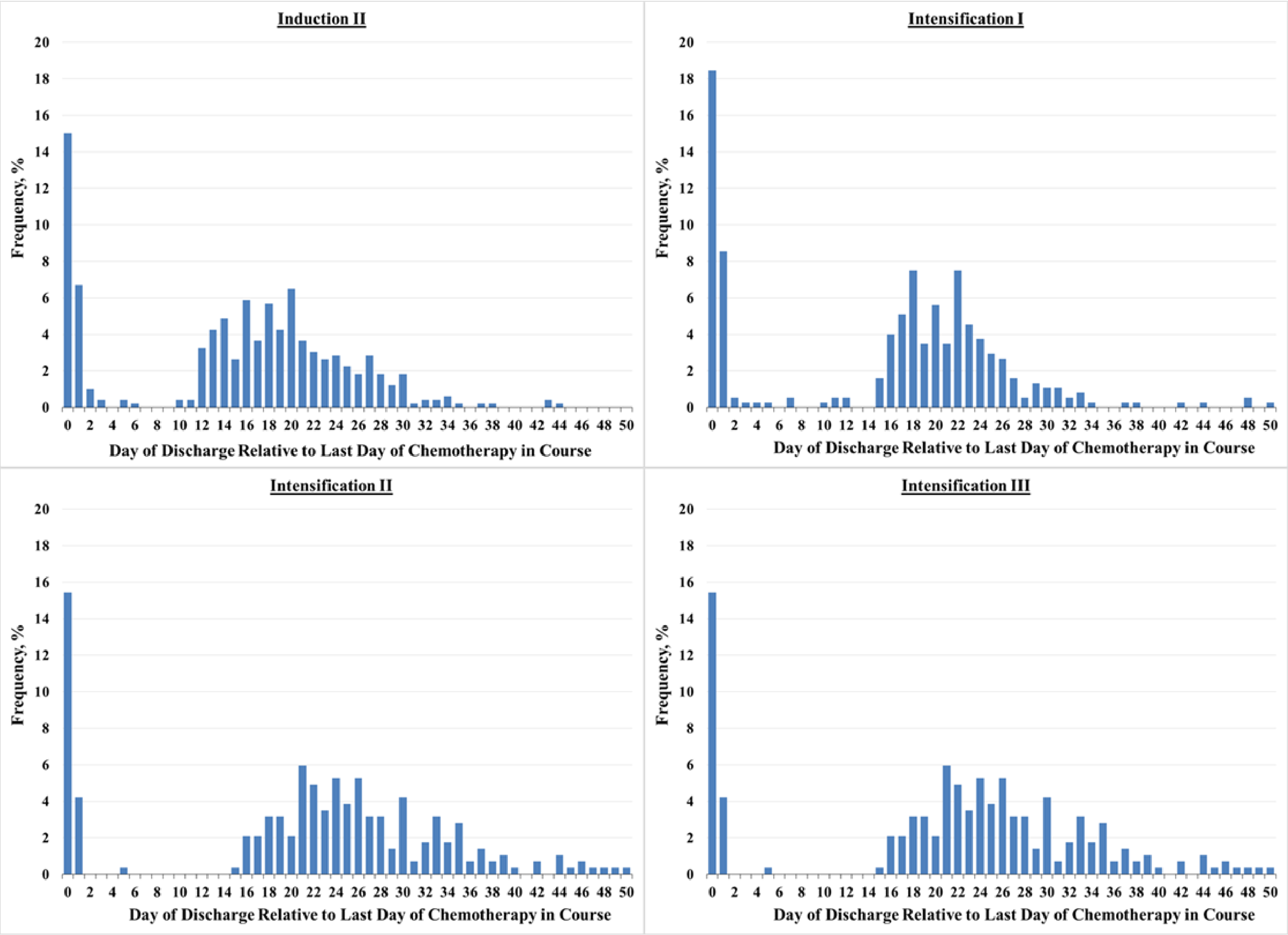

Patients discharged with 3 days after the last chemotherapy administration in a given course where classified as Outpatient Management.

© 2021 Getz KD et al. *JAMA Network Open.*

eFigure 3. Flowchart Depicting the Study Population Completing HRQOL and Each of the Secondary Patient-Centered Outcome Assessments

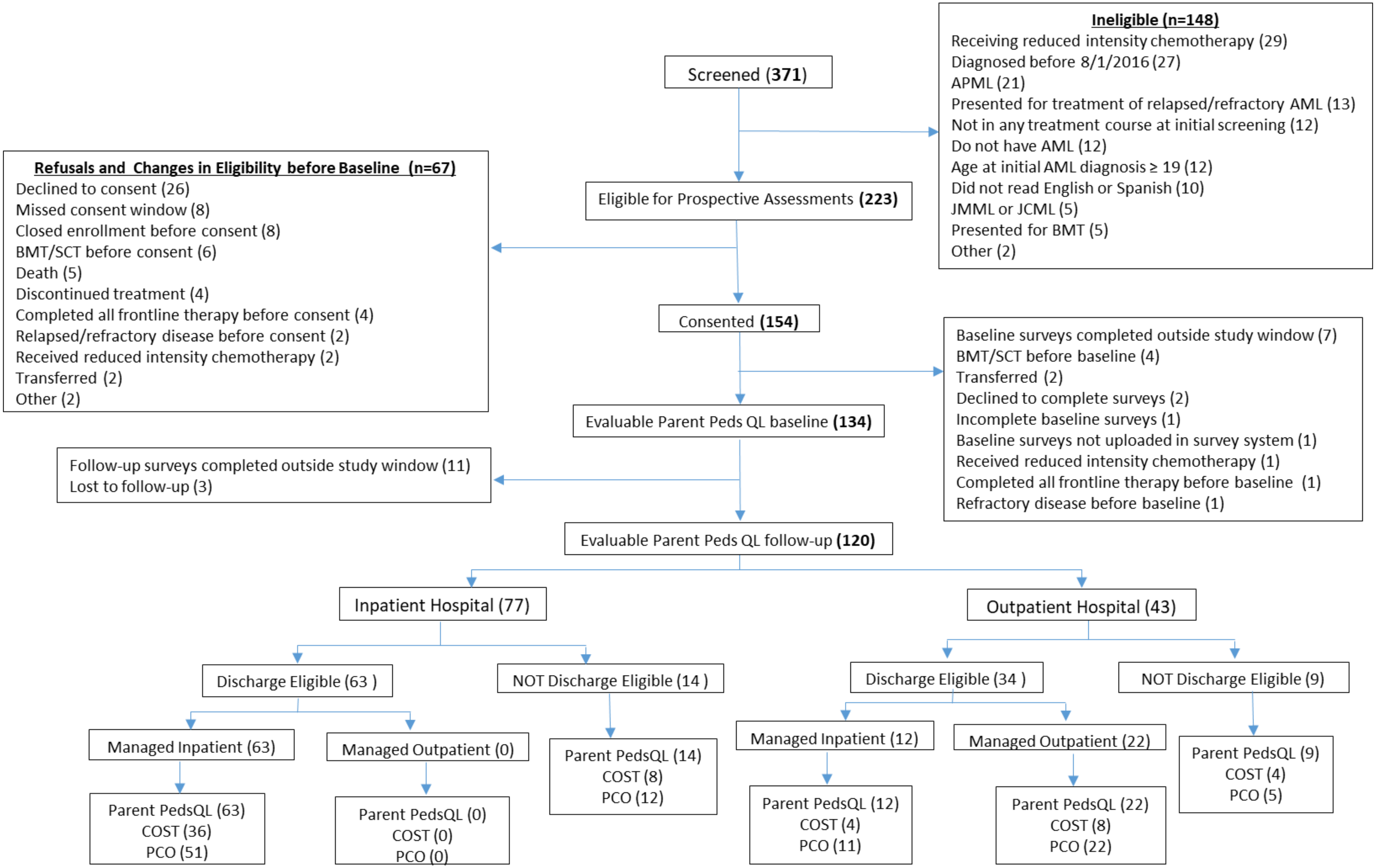

© 2021 Getz KD et al. *JAMA Network Open.*
